# Fat-targeted small molecule alleviates abnormal adipose tissue remodeling in obesity via SIRT3-driven mitophagy and inflammasome inhibition

**DOI:** 10.1186/s13020-025-01253-4

**Published:** 2025-12-10

**Authors:** Kegang Linghu, Longkun Hu, Yu-E. Wang, Yuxia Zhou, Yuanyuan Wang, Mingjun Shi, Lirong Liu, Hua Yu, Lei Tang, Ligen Lin, Bing Guo, Ai Tian, Tian Zhang

**Affiliations:** 1 Guizhou Provincial Key Laboratory of Pathogenesis and Drug Research On Common Chronic Diseases, Guizhou Medical University, Guiyang, 550025 Guizhou China; 2 Guizhou Institute of Precision Medicine, Affiliated Hospital of Guizhou Medical University, Guiyang, 550025 Guizhou China; 3 State Key Laboratory of Discovery and Utilization of Functional Components in Traditional Chinese Medicine, Guizhou Medical University, Guiyang, 550025 Guizhou China; 4 State Key Laboratory of Quality Research in Chinese Medicine, Institute of Chinese Medical Sciences, University of Macau, Macau, China; 5 The Affiliated Stomatological Hospital &, Stomatology of Guizhou Medical University, Guiyang, 550025 Guizhou China

**Keywords:** Abnormal adipose tissue remodeling, Alpha lipoamide, Autophagy, Obesity, Nano-emulsion, NLRP3 inflammasome

## Abstract

**Background:**

In obesity, excessive energy intake and the expansion of adipose tissue increase ROS generation, contributing to adipocyte dysfunction and inflammation, which leads to abnormal adipose tissue remodeling (ATR). Alpha lipoamide (ALM) is the neutral amide form of lipoic acid, a natural antioxidant extracted from plant-based foods such as asparagus, spinach, and broccoli. This work focuses on ALM's beneficial effects and mechanism in adipose tissue inflammation (ATI) and abnormal ATR in obesity.

**Methods:**

The anti-inflammatory effect of ALM was evaluated by ELISA, flow cytometry, Western blots, and immunofluorescence assays. The binding affinity of ALM to SIRT3 deacetylase was evaluated through cellular thermal shift assay (CETSA) and molecular docking. The adipose tissue-targeting alpha lipoamide nanoemulsion (ALM-NE) was validated using small animal live imaging. Adipose tissue inflammation was evaluated by histological analysis and immunohistochemical staining in both high-fat diet (HFD) and LPS plus ATP-induced inflammation models in mice.

**Results:**

ALM suppressed the activation of NLRP3 inflammasome via enhancing SIRT3-mediated autophagy. Co-immunoprecipitation revealed that ALM blunted mitochondrial damage through SIRT3-mediated SOD2 deacetylation and FUNDC1-mediated mitophagy activation, resulting in ROS reduction and NLRP3 inflammasome inactivation. Moreover, ALM mitigates inflammatory crosstalk between macrophages and adipocytes in an in vitro co-culture model. Finally, we established an adipose tissue-targeting ALM-NE, which alleviated ATI in LPS and ATP-induced acute inflammation in mice and inhibited abnormal ATR in high-fat diet-induced obese mice.

**Conclusion:**

In summary, ALM attenuates inflammatory crosstalk between M1 macrophages and adipocytes by enhancing SIRT3-mediated mitophagy and suppressing NLRP3 inflammasome activation, thereby alleviating adipose tissue inflammation and pathological remodeling in obesity. Thus, ALM has the capacity to become a therapeutic candidate for treating obesity and its associated metabolic disorders.

**Graphical Abstract:**

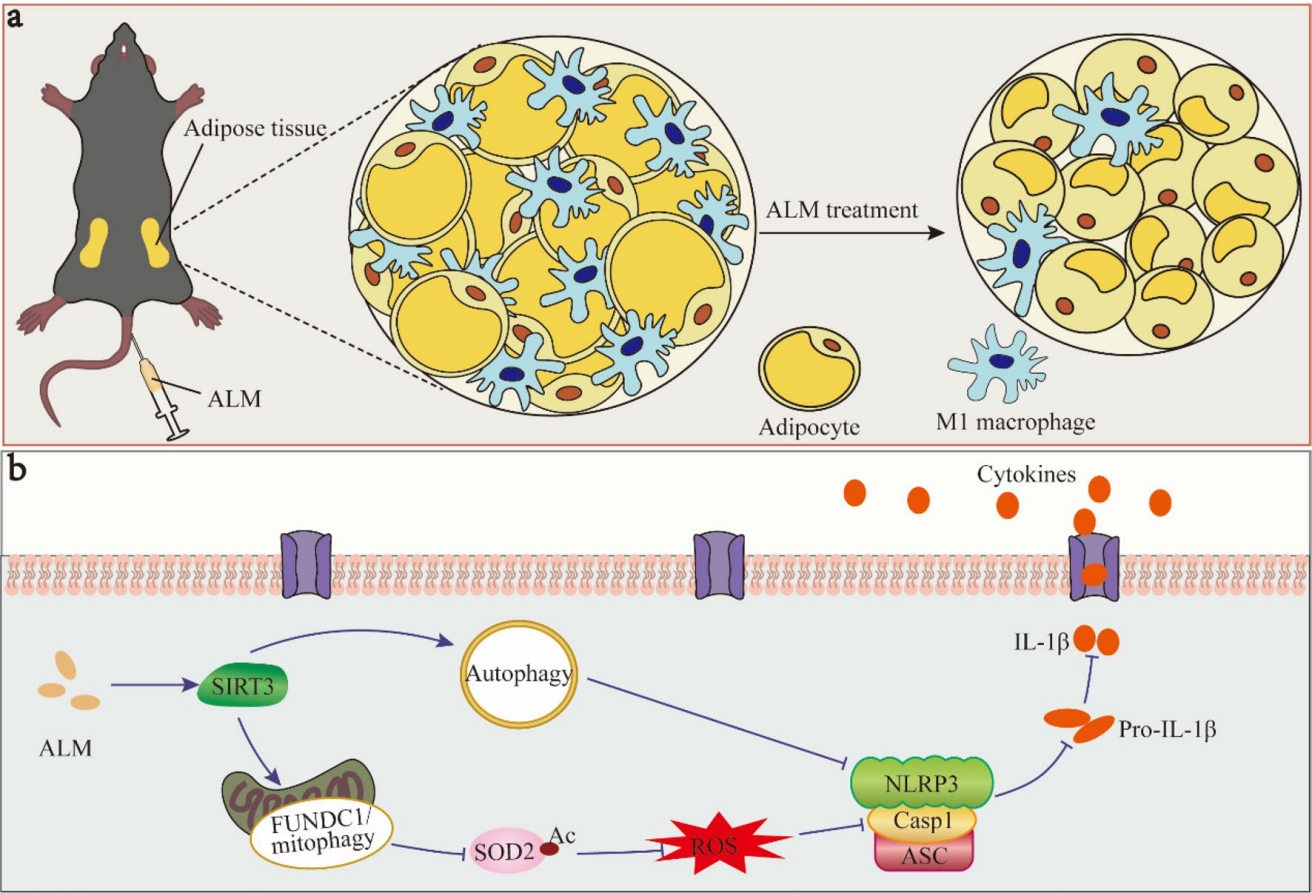

**Supplementary Information:**

The online version contains supplementary material available at 10.1186/s13020-025-01253-4.

## Introduction

In obesity, adipose tissue undergoes abnormal remodeling (hypertrophy; enlargement of fat cells) and hyperplasia (increased fat cell number) in response to excess energy surplus [1]. Excess energy intake and abnormal adipose tissue expansion initiate a pro-inflammatory state. Inflammation, in turn, disrupts adipose tissue function, creating a vicious cycle that exacerbates weight gain and insulin resistance [2]. In the obese adipose tissue, immune cells, particularly macrophages, increase significantly. These macrophages shift from an anti-inflammatory (M2) phenotype in lean tissue to a pro-inflammatory (M1) phenotype, exacerbating inflammation. This phenoyepic switch amplifies local inflammation through cellular interaction between adipocytes and macrophages, driving both adipose tissue inflammation (ATI) and abnormal adipose tissue remodeling (ATR) [3]. Therefore, suppressing M1 macrophages may provide a key mechanism for inhibiting adipose ATI and abnormal ATR.

The Nod-like receptor (NLR) family pyrin domain-containing 3 (NLRP3) protein complex comprises numerous units that stimulate the innate immune response. The structure consists of three domains: the amino-terminal pyrin domain (PYD), the central nucleotide-binding domain (NOD), and the leucine-rich-repeat (LRR). The initiation of NLRP3 inflammasome triggers the auto-cleavage and activation of pro-caspase-1, leading to the maturation and secretion of the pro-inflammatory cytokines IL-1β and IL-18 [4, 5]. Autophagy is an evolutionarily conserved process that includes the breakdown of intracellular components and is crucial for constraining the NLRP3 inflammasome activation [6, 7]. Studies have shown that genetic deletion of autophagy-related protein 5 (ATG5) aggravated lipopolysaccharides (LPS) -induced inflammatory response in mice [8]. Similarly, macrophage-specific knockdown of ATG5 enhanced the release of mature-IL-1β in silica-induced acute lung inflammation [9]. Consistently, autophagy induction suppressed NLRP3 inflammasome activation in macrophages [10, 11]. Thus, autophagy-mediated suppression of the NLRP3 inflammasome is a potential strategy for inhibiting inflammatory conditions.

In obesity, adipose tissue is characterized by mitochondrial dysfunction and endoplasmic reticulum stress [12, 13]. Mitophagy, a selective form of autophagy, plays a crucial role in maintaining mitochondrial homeostasis by targeting the selective removal of damaged mitochondria. Impairment of mitophagy results in the accumulation of dysfunctional, ROS-generating mitochondria, which exacerbates oxidative stress and promotes disease pathogenesis [13, 14]. Emerging evidence suggests that defective mitophagy contributes significantly to NLRP3 inflammasome activation [15, 16]. Notably, FUNDC1—a key mitophagy receptor—has been shown to mitigate NLRP3-driven inflammation in mice by enhancing mitophagic flux [17, 18]. Therefore, enhancing mitophagy represents a promising therapeutic strategy to suppress inflammation and preserve mitochondrial integrity.

SIRT3 (sirtuin 3), a mitochondrial NAD + dependent deacetylase in the mitochondrial matrix, was essential in regulating mitochondrial energy metabolism and homeostasis [12, 13]. SIRT3 has been reported to improve mitochondrial function via mitophagy-mediated ROS clearance. SIRT3 activates mitophagy, which removes damaged mitochondria, reducing the sources of excessive ROS production [14, 15]. More importantly, SIRT3 can directly bind and deacetylate SOD2, increasing SOD2 activity, positively affecting mtROS homeostasis, and inhibiting NLRP3 inflammasome activation [16, 17]. This prevents a vicious cycle in which ROS-induced mitochondrial damage leads to further generation of ROS. Therefore, SIRT3 is a druggable target for mtROS homeostasis and inhibiting NLRP3 inflammasome activation.

Alpha lipoamide (ALM) is the neutral amide form of lipoic acid (LA) which is a natural antioxidant extracted from plant-based foods such as asparagus, spinach, and broccoli. Importantly, ALM exhibits superior efficacy compared to lipoic acid (LA) in attenuating mitochondrial dysfunction and oxidative stress [18, 19]. Our previous research indicates that ALM mitigates renal fibrosis by curbing high-glucose-induced inflammation and improving the antioxidant capacity of renal tubular epithelial cells, thus preventing the fibrosis in these cells under high-glucose conditions [20, 21]. Additionally, Zhao et al. discovered that ALM improves diabetic nephropathy by inhibiting reactive oxygen species (ROS) generation and enhancing mitochondrial function [22]. These findings highlight the therapeutic potential of ALM on metabolic diseases. In the present study, we investigated the effects of ALM on ATI and abnormal ATR. Our results showed that ALM repaired impaired mitophagy, leading to a restoration of mitochondrial membrane potential and a reduction in the mitochondrial reactive oxygen species (mtROS) levels, thereby suppressing NLRP3 inflammasome activation. Importantly, SIRT3 was demonstrated as the direct target of ALM; ALM initiated the autophagy and suppressed the activation of NLRP3 inflammasome through a direct activation of SIRT3. Furthermore, we developed an adipose tissue-targeting ALM nano-emulsion (ALM-NE), which attenuated obesity via alleviating ATI and suppressing abnormal ATR.

## Results

### ALM inhibited LPS plus adenosine 5′-triphosphate (ATP)-induced oxidative stress and inflammatory response in RAW264.7 macrophages

**Fig. 1:**
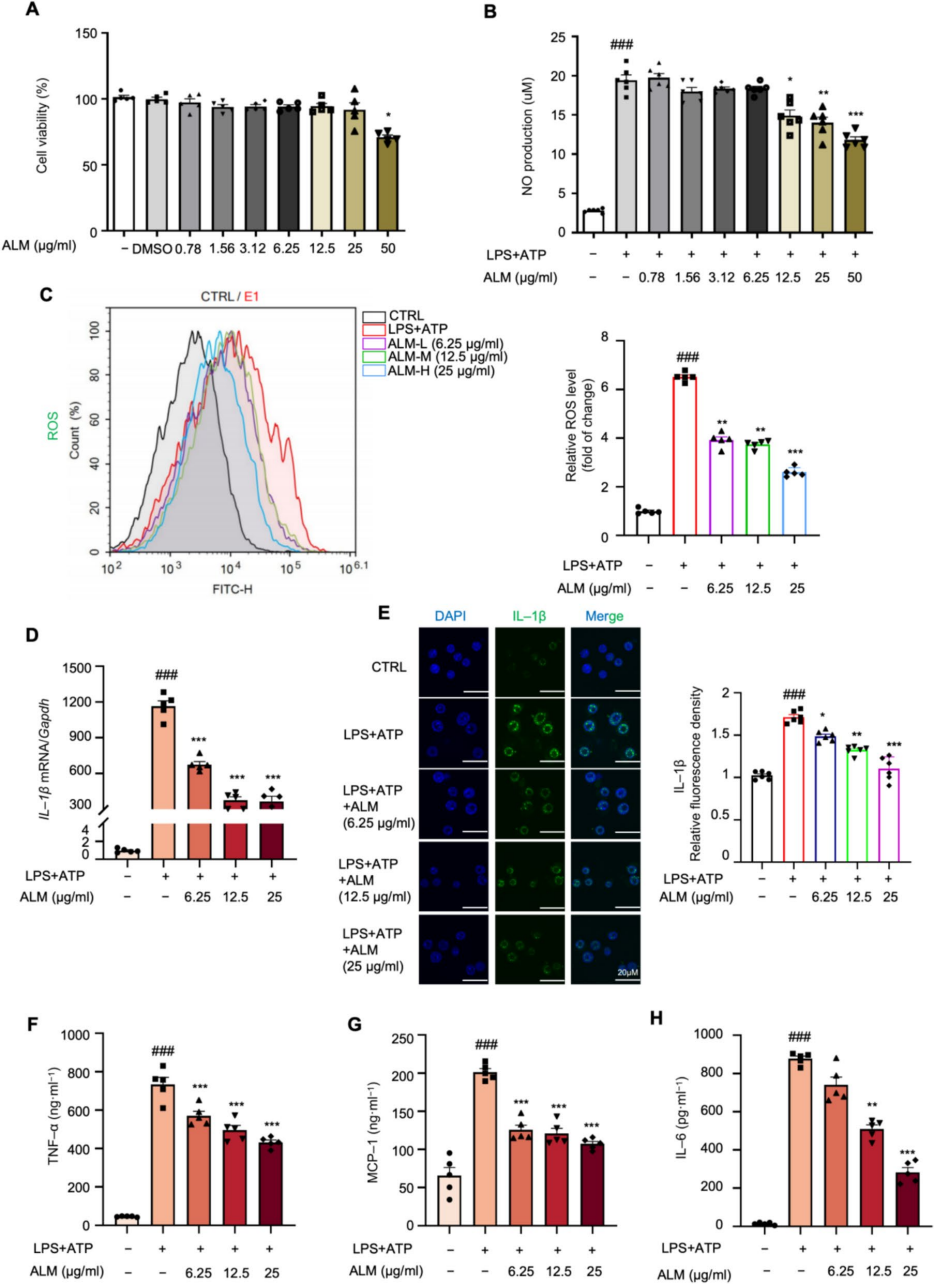
ALM inhibited LPS plus adenosine 5′-triphosphate (ATP)-induced oxidative stress and inflammatory response in RAW264.7 macrophages. Raw264.7 were treated with various concentrations of ALM and challenged with or without LPS + ATP for 18 h. **A** Cell viability assay of RAW264.7 cells treated with different concentrations of ALM by MTT assay. (*n* = 6). **B** Nitric oxide (NO) production was analyzed in LPS plus ATP-challenged macrophages treated with different concentrations of ALM by Griess reagent (*n* = 6). **C** The level of intracellular reactive oxygen species (ROS) of macrophages was analyzed by flow cytometry (*n* = 5). **D** Quantitative real-time PCR analysis of *Il-1β* mRNA level in LPS plus ATP-stimulated macrophages with the indicated concentration of ALM (*n* = 5). **E** Immunofluorescence staining of IL-1β in LPS plus ATP-stimulated macrophages with the indicated concentration of ALM (*n* = 5). **F**–**H** Measurement of the TNF-α, MCP-1, and IL-6 levels in the culture medium by ELISA kits (*n* = 5). Data are expressed as means ± SEM. ^#^*P* < 0.05, ^##^*P* < 0.01, ^###^*P* < 0.001, LPS + ATP vs. CTRL; *P < 0.05, **P < 0.01, ***P < 0.001, LPS + ATP + ALM vs. LPS + ATP

Inflammation and oxidative stress are interdependent processes that mutually reinforce each other in obesity [23]. ALM has been reported for its excellent antioxidant activity [20, 22]. Thus, we characterized the effect of ALM on inflammatory suppression in the LPS plus ATP-stimulated inflammatory RAW264.7 macrophages. Under the non-cytotoxic concentrations, ALM inhibited nitric oxide release and intracellular reactive oxygen species (ROS) production in a dose-dependent manner (Figs. 1A–C and S1A). ALM significantly downregulated the mRNA expression of *IL-1β* in macrophages following LPS plus ATP treatment (Fig. 1D). The immunostaining results consistently offered additional support for this conclusion (Fig. 1E). In addition, ALM exhibited a dose-dependent hindrance in TNF-α, MCP-1, and IL-6 levels in macrophages activated by LPS and ATP (Figs. 1F–H). The findings demonstrate that ALM effectively inhibited the oxidative stress and inflammatory response in RAW264.7 macrophages induced by LPS plus ATP.

### ALM attenuated NLRP3 inflammasome activation by enhancing autophagy

**Fig. 2:**
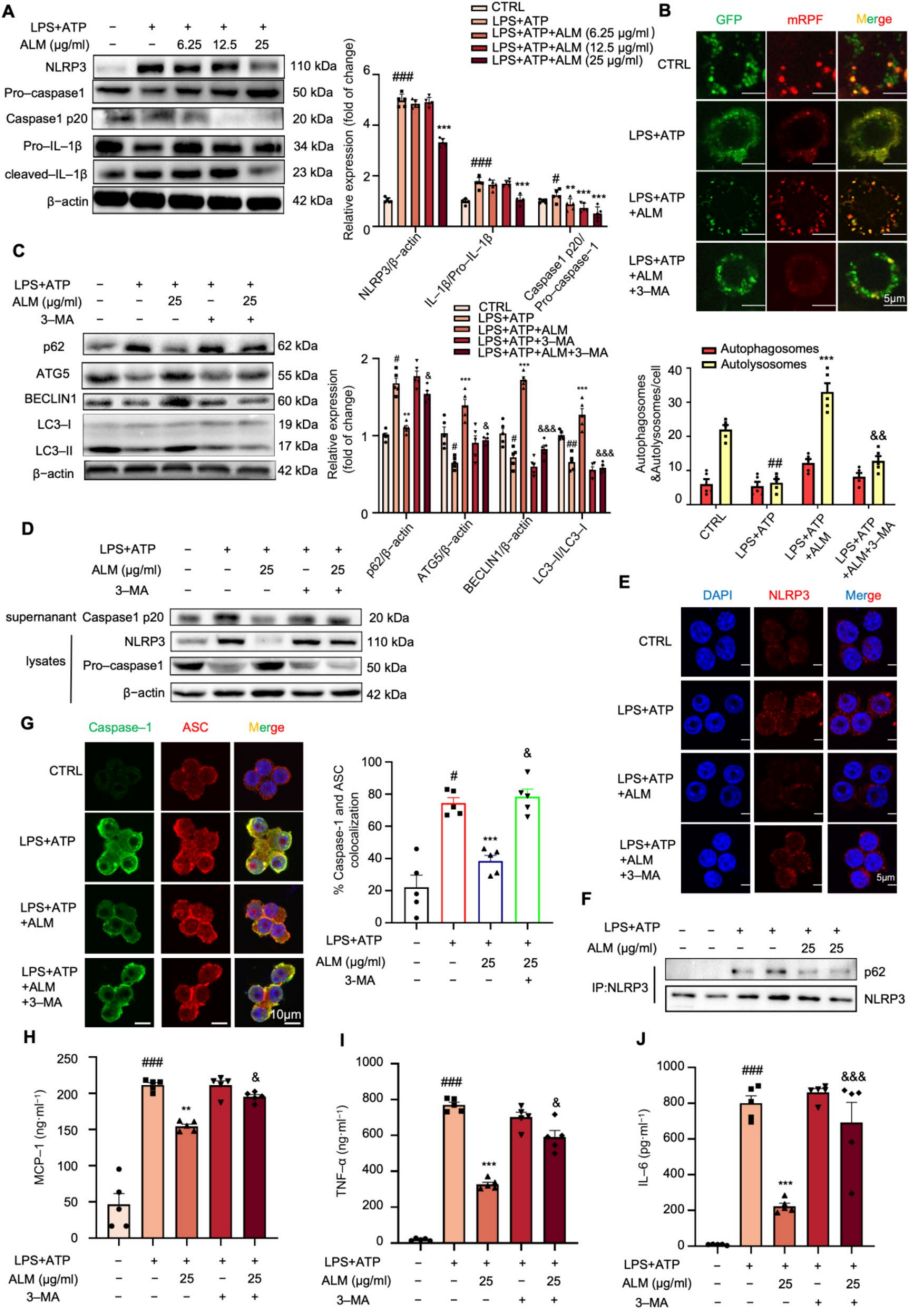
ALM attenuated NLRP3 inflammasome activation by enhancing autophagy. **A** Western blots of NLRP3, Caspase-1, and IL-1β in the lysates of RAW264.7 cells (*n* = 5). β-actin was used as an internal loading control. **B** RAW264.7 cells were transiently infected with the mRFP-GFP-LC3 lentivirus for 24 h. Then, the cells were subjected to the indicated treatments. mRFP-GFP-LC3 puncta were measured using a confocal microscope (*n* = 5). **C** Western blot analysis of autophagy-related proteins was measured in LPS plus ATP-stimulated Raw264.7 cells with different concentrations of ALM. (*n* = 5). β-actin was used as an internal loading control. **D** Western blot analysis of NLRP3 and pro-caspase-1 in the lysates and cleaved caspase-1 in the supernatant (*n* = 5). **E** Immunofluorescence staining of NLRP3 (red) was performed in LPS plus ATP-stimulated Raw264.7 macrophages with the indicated concentration of ALM and 5 mM 3-MA (*n* = 5). **F** Co-immunoprecipitation assay of p62 with NLRP3 in LPS plus ATP and ALM-treated RAW264.7 cells (*n* = 5). **G** Immunofluorescence staining of caspase-1 and ASC was performed in LPS plus ATP-stimulated Raw264.7 cells with the indicated concentration of ALM and 5 mM 3-MA (*n* = 5). **H**–**J** The levels of MCP-1, TNF-α, and IL-6 in the culture medium were determined by ELISA kits (*n* = 5). Data are expressed as means ± SEM. ^#^*P* < 0.05, ^##^*P* < 0.01 and ^###^*P* < 0.001, LPS + ATP vs. CTRL; *P < 0.05, **P < 0.01 and ***P < 0.001, LPS + ATP + ALM vs. LPS + ATP; ^&^*P* < 0.05, ^&&^*P* < 0.01 and ^&&&^*P* < 0.001, LPS + ATP + ALM vs. LPS + ATP + ALM. + 3-MA

The NLRP3 inflammasome primarily contributes to the maturation and secretion of IL-1β [24]. Consistent with our expectations, the ALM treatment suppressed the expression of NLRP3 and the activity of caspase-1 and IL-1β in RAW264.7 macrophages stimulated by LPS and ATP (Fig. 2A). The immunostaining findings consistently supported the above observation (Fig. S1B). Interestingly, ALM treatment exhibited a dose-dependent increase in ATG5 and BECN1 levels, as well as a rise in the LC3-II to LC3-I ratio, and a reduction in p62 (Fig. S1C). To further determine whether ALM increases autophagic flux, RAW264.7 macrophages were infected with mRFP-GFP-LC3 adenovirus. As shown in Fig. 2B, a higher number of red-only puncta and a smaller number of yellow puncta in macrophages treated with ALM suggested that ALM enhanced autophagic flux by promoting undisturbed lysosomal function and/or autophagosome-lysosome fusion. Importantly, the pro-autophagic effects of ALM were abolished by 3-MA (an autophagy inhibitor) co-treatment (Figs. 2B, C). Consistently, the inhibitory effect of ALM on NLRP3 inflammasome activation was reversed by 3-MA (Figs. 2D–E and S1D–E).

To further investigate the involvement of ALM-driven autophagy in NLRP3 inflammasome inactivation, the combination of NLRP3 and autophagy components was detected. The co-immunoprecipitation assays showed an increased p62 content was pulled down by the same amount of NLRP3 antibody following LPS and ATP administration, which was significantly attenuated by ALM treatment (Fig. 2F), suggesting that ALM promotes the autophagic degradation of the p62-NLRP3 immune complex. Furthermore, we conducted the immunofluorescence labeling to determine the colocalization of caspase-1 and ASC. As shown in Fig. 2G, ALM disrupted the formation of the ASC-caspase-1 complex in LPS and ATP activated macrophages, while 3-MA cotreatment reversed these trends. Consistent with these findings, 3-MA also reversed the ALM-mediated reduction in inflammatory cytokines (Figs. 2H–J), underscoring the crucial role of autophagy in ALM-mediated inactivation of NLRP3 inflammasome.

### *ALM promoted autophagy *via* targeting and increasing SIRT3 deacetylase activity*

**Fig. 3:**
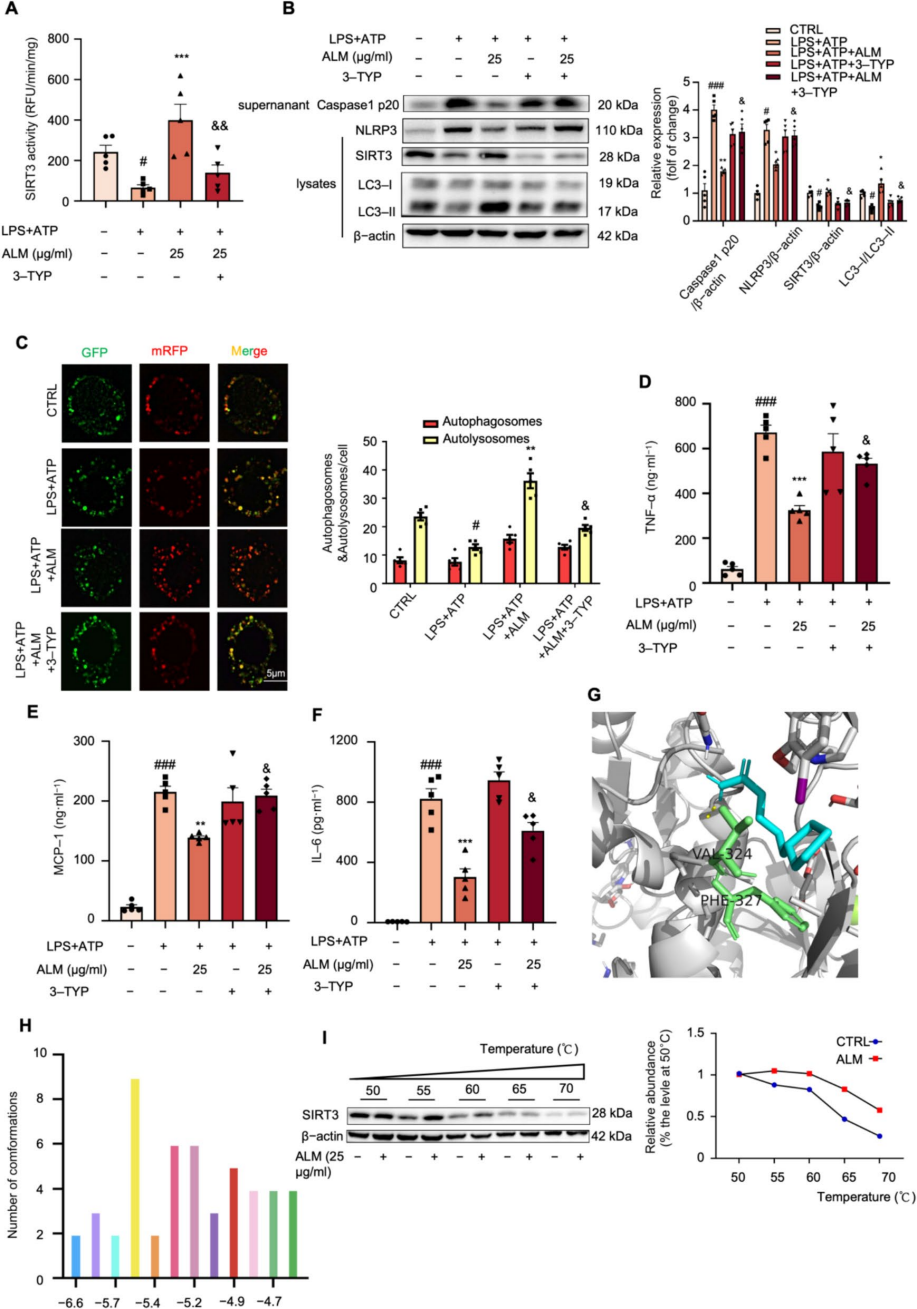
ALM promoted autophagy via targeting and increasing SIRT3 deacetylase activity. **A** Deacetylase activity of SIRT3 was detected in LPS plus ATP-stimulated Raw264.7 with the indicated concentration of ALM and 50 μM 3-TYP (*n* = 5). **B** Western blot analysis of the expression of NLRP3, Caspase1 p20, SIRT3, and LC3 protein levels in LPS plus ATP-stimulated Raw264.7 with the indicated concentration of ALM and 50 μM 3-TYP (*n* = 5). β-actin was used as an internal loading control. **C** RAW264.7 cells were transiently infected with the mRFP-GFP-LC3 lentivirus for 24 h. mRFP-GFP-LC3 puncta were measured using a confocal microscope (*n* = 5). **D**–**F** The cytokine levels of TNF-α, MCP-1, and IL-6 in the culture medium were determined by ELISA kits (*n* = 5). **G** Interaction between ALM and residues on SIRT3. **H** Docking results of the binding between ALM and SIRT3 (PBD ID:5H4D). Cluster analysis of the docked conformations of ALM. A tolerance of 2.0 Å was used. **I** Cellular thermal shift assay (CETSA) was performed on RAW264.7 cells after the treatment with or without ALM (25 μg·ml^−1^) for 18 h (*n* = 5). Data are expressed as means ± SEM. ^#^*P* < 0.05 and ^###^*P* < 0.001, LPS + ATP vs. CTRL; ^###^ *P* < 0.01 and *** *P* < 0.001, LPS + ATP + ALM vs. LPS + ATP; & *P* < 0.05, LPS + ATP + ALM vs. LPS + ATP + ALM + 3-TYP

Sirtuin 3 (SIRT3)-deficient macrophages displayed hindered autophagy and heightened activation of the NLRP3 inflammasome [25]. Interestingly, ALM treatment increased SIRT3 deacetylase activity in LPS and ATP-challenged RAW264.7 cells (Fig. 3A). Moreover, ALM-driven inactivation of NLRP3 inflammasome and increase of the ratio of LC3-II to LC3-I was reversed by co-administration of 3-TYP (a proven specific inhibitor of SIRT3) (Fig. 3B). The fluorescence findings of mRFP-GFP-LC3 consistently demonstrated that the combination of ALM and 3-TYP decreased the presence of red puncta, indicating a disruption in the autophagic flux (Fig. 3C). In addition, the ALM-induced suppression of TNF-α, MCP-1 and IL-6 cytokine levels in RAW264.7 cells were counteracted via co-treatment with 3-TYP (Figs. 3D–F). To further validate the role of Sirt3, we carried out shRNA-mediated Sirt3 knockdown (Sirt3KD) in RAW264.7 macrophages. The results demonstrated the genetic inactivation of SIRT3 abolished the beneficial effects of ALM, as shown by a significant reversal of ALM-driven NLRP3 inflammasome inactivation, suppression of autophagy activation, and instigated the pro-inflammatory cytokine levels (Figs S2A-H). Collectively, ALM facilitated the deactivation of the NLRP3 inflammasome by increasing SIRT3 deacetylase activity and enhancing autophagy.

To investigate the potential mechanisms by which ALM activated SIRT3, we performed molecular docking analysis to evaluate the binding interaction between ALM and SIRT3. The results showed that ALM had a hydrophobic interaction with amino acid residues (VAL324, PHE327) in the SIRT3 binding pocket (Fig. 3G). Clustering analysis showed the best binding pose in the predominant cluster (the yellow bar, Fig. 3H), which possessed the binding energy score of − 5.5 kCal/mol, suggesting a stable interaction. Furthermore, cellular thermal shift assay (CETSA) results suggested that ALM treatment stabilized the SIRT3 protein compared to the control cells (Fig. 3I). Notably, ALM exhibited no appreciable binding to the other SIRT family proteins (Figs. S2J–Q), highlighting its selectivity for SIRT3. Taken together, the results suggest that SIRT3 could be the druggable target of ALM in activating autophagy and inhibiting NLRP3 inflammasome.

### ALM blunted mitochondrial damage through SIRT3-mediated SOD2 deacetylation and mitophagy activation

**Fig. 4:**
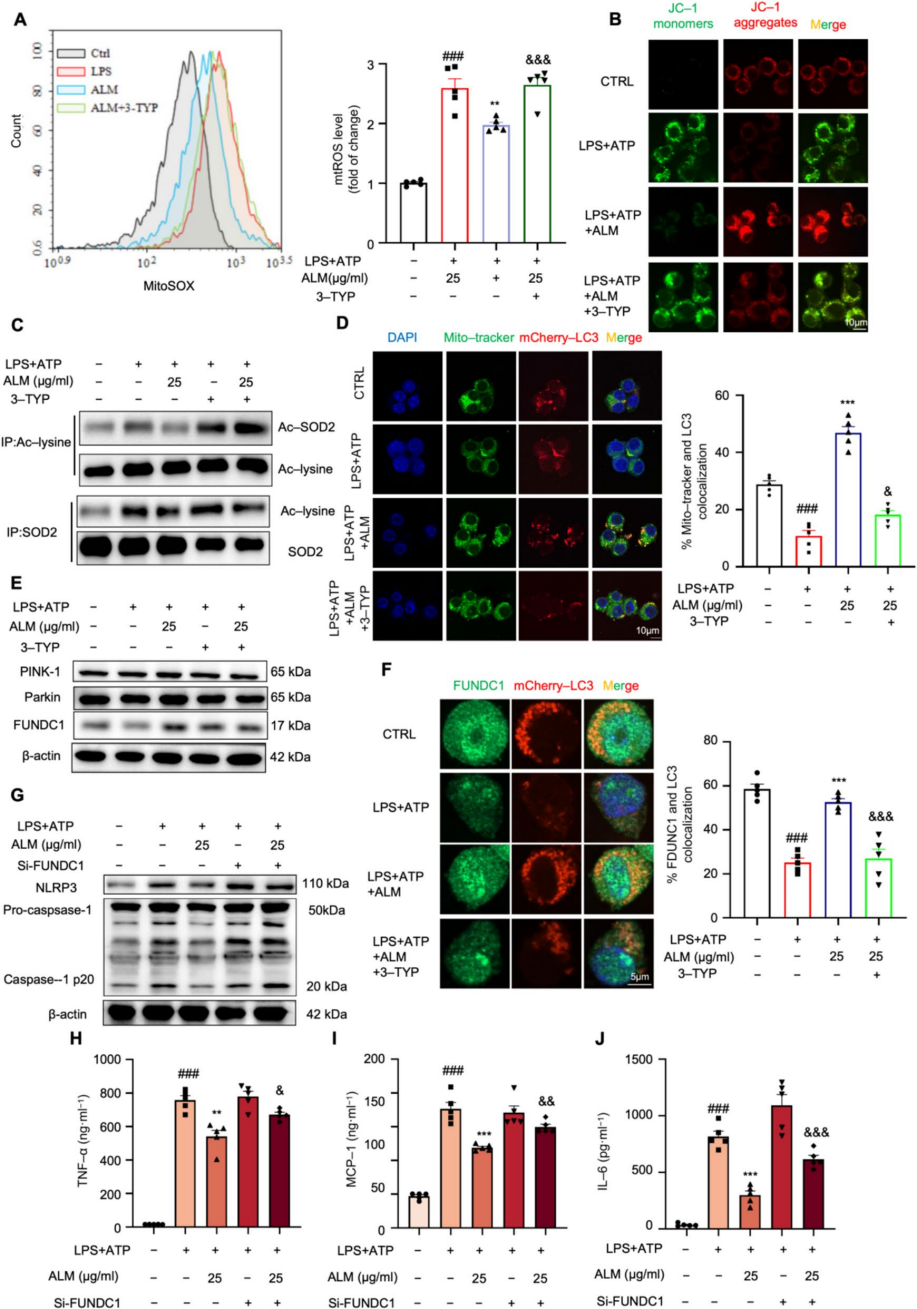
ALM blunted mitochondrial damage through SIRT3-mediated SOD2 deacetylation and mitophagy activation. **A** The mitochondrial reactive oxygen species (mtROS) level was determined by MitoSOX in LPS plus ATP-challenged RAW 264.7 cells treated with or without ALM and 3-TYP (*n* = 5). **B** Mitochondrial membrane potential was evaluated by JC-1 staining (*n* = 6). **C** Co-IP assay indicates the level of acetylated SOD2 in LPS plus ATP-challenged RAW 264.7 cells treated with or without ALM and 3-TYP (*n* = 5). **D** RAW264.7 cells were transiently infected with the mCherry-LC3 plasmid for 24 h, and immunofluorescence staining of Mito-tracker was performed (*n* = 5). **E** Western blot analysis of mitophagy components PINK1, Parkin, and FUNDC1 in LPS plus ATP-stimulated macrophages with the indicated concentration of ALM and 3-TYP (*n* = 5). β-actin was used as an internal loading control. **F** The immunofluorescence staining of FUNDC1 and mCherry-LC3 was performed (*n* = 5). Data are expressed as means ± SEM. **G** Western blot analysis of NLRP3 and Caspase1 p20 levels in LPS plus ATP-stimulated macrophages with the indicated concentration of ALM and Si-FUNDC1 (*n* = 5). β-actin was used as an internal loading control (*n* = 5). **H–J** The levels of TNF-α, MCP-1, and IL-6 in the culture medium were determined by ELISA kits (*n* = 5). Data are expressed as means ± SEM. ^#^*P* < 0.05 and ^###^*P* < 0.001, LPS + ATP vs. CTRL; *P < 0.05, **P < 0.01 and ***P < 0.001, LPS + ATP + ALM vs. LPS + ATP; ^&^*P* < 0.05 and ^&&^*P* < 0.01, LPS + ATP + ALM vs. LPS + ATP + ALM + 3-TYP or LPS + ATP + ALM + Si-FDUNC1

Mitochondrial perturbation could trigger the release of mitochondrial reactive oxygen species (mtROS), which in turn promotes the assembly of the NLRP3 inflammasome complex [26, 27]. Thus, we conducted further investigations to determine whether ALM hindered the activation of NLRP3 inflammasome by mitigating mitochondrial damage. As shown in Fig. 4A, ALM attenuated LPS plus ATP-induced mtROS accumulation. The JC-1 staining assay showed a pronounced green fluorescence shift as the mitochondria became depolarized in LPS plus ATP-treated macrophages, and the green fluorescence was significantly hindered by treatment with ALM (Figs. 4B and S3A). Co-immunoprecipitation results demonstrated that ALM scavenges mitochondrial-derived ROS by reducing SOD2 acetylation levels (Fig. 4C). These protective effects were reversed by co-treatment with 3-TYP, which indicates that ALM blunted mitochondrial damage through SIRT3-dependent SOD2 deacetylation.

Furthermore, ALM promoted autophagosomes to relocate damaged mitochondria to adopt a dispersed distribution (Fig. 4D), suggesting that ALM might activate mitophagy. However, ALM did not significantly alter the expression of PINK1 or Parkin (Fig. 4E), implying the involvement of alternative pathways in ALM-SIRT3-mediated mitophagy in macrophages. Instead, we found that FUN14 domain-containing protein 1 (FUNDC1), a new mitophagy receptor, participated in the ALM-driven mitophagy (Figs. 4E–F). Notably, loss of FUNDC1 abolished the beneficial role of ALM in NLRP3 inflammasome activation (Fig. 4G). The inhibitory effects of ALM on pro-inflammatory cytokines were also abrogated after loss of FUNDC1 (Figs. 4H–J). These results indicate that ALM restores mitochondrial homeostasis by enhancing FUNDC1-mediated mitophagy in a SIRT3-dependent manner, counteracting mitochondrial disruption in LPS plus ATP-treated macrophages.

### ALM suppressed inflammatory crosstalk between macrophages and adipocytes

**Fig. 5:**
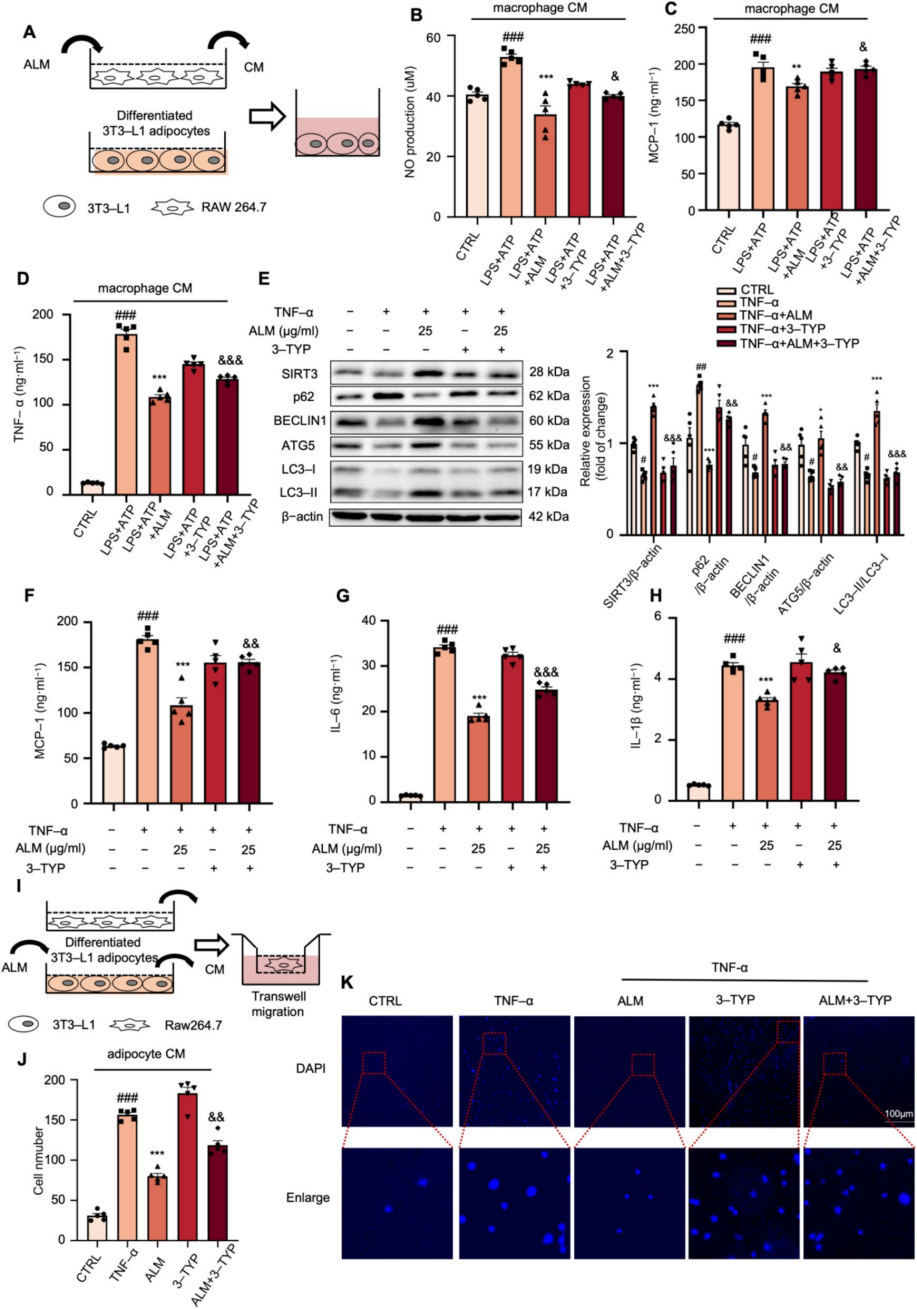
ALM suppressed inflammatory crosstalk between macrophages and adipocytes. **A** The experimental procedures of the macrophage-adipocyte co-culture experiments. Fully differentiated 3T3-L1 were incubated with macrophage-conditioned media (CM) for 24 h. **B**–**D** The NO production and the levels of MCP-1 and TNF-α in 3T3-L1 adipocytes were determined (*n* = 5). Fully differentiated 3T3-L1 adipocytes were treated with 25 μg·ml-1 ALM and TNF-α (15 ng·ml^−1^) for 24 h, followed by stimulation with 50 μM 3-TYP for 6 h. **E** Western blot analysis of autophagy-related proteins in TNF-α-treated adipocytes with the indicated concentration of ALM and 3-TYP (*n* = 5). β-actin was used as an internal loading control. **F**–**H** The levels of MCP-1, IL-6, and IL-1β in the culture medium from 3T3-L1 adipocytes were examined by ELISA kits (*n* = 5). **I** The procedures of the macrophage migration experiments. Fully differentiated 3T3-L1 adipocytes were treated with 25 μg·ml^−1^ ALM and TNF-α (15 ng·ml^−1^) for 24 h, followed by stimulation with 50 μM 3-TYP for 6 h. Then, the cells were changed to a fresh medium. After 24 h, the medium supernatants were collected as adipocyte CM. RAW264.7 cells were incubated in adipocyte CM for 4 h (*n* = 5). **J**, **K** Transwell assay of the migrated macrophages was quantified using DAPI staining. Data are expressed as means ± SEM. ^#^*P* < 0.05, ^##^*P* < 0.01 and ^###^*P* < 0.001, macrophage CM + LPS + ATP vs. macrophage CM + CTRL; TNF-α vs. CTRL; *P < 0.05 and ***P < 0.001, macrophage CM + LPS + ATP + ALM vs. macrophage CM + LPS + ATP,; TNF-α + ALM vs. TNF-α; ^&^*P* < 0.05, ^&&^*P* < 0.01 and ^&&&^*P* < 0.001, macrophage CM + LPS + ATP + ALM vs. macrophage CM + LPS + ATP + ALM + 3-TYP; TNF-α + ALM vs. TNF-α + ALM + 3-TYP

To investigate the impact of cytokines produced by macrophages on adipocytes. We exposed differentiated 3T3-L1 adipocytes to conditional medium (CM) obtained from RAW264.7 macrophages that were treated with or without ALM (Fig. 5A). The inflammatory macrophage-derived CM significantly elevated the generation of NO, MCP-1, and TNF-α in adipocytes. At the same time, CM from macrophages pretreated with ALM restored these alterations, and the beneficial effects of ALM were counteracted by co-treatment with 3-TYP (Figs. 5B–D). The above results suggested that ALM ameliorated macrophage CM-induced inflammatory responses in adipocytes.

The activation of inflammation in adipocytes is critically influenced by TNF-α generated by macrophages. Hence, the anti-inflammatory impact of ALM was assessed in adipocytes stimulated with TNF-α. Stimulation with TNF-α results in a significant downregulation of SIRT3, BECLIN1, and ATG5 protein levels and a decrease in the LC3-II/LC3-I ratio (ranging from 44 to 70% compared with the control group), while p62 levels rose to 150% compared to the control cells (Fig. 5E). This suggests that autophagy in TNF-α-stimulated adipocytes is compromised. ALM pretreatment altered these patterns, but the co-treatment of 3-TYP partially reversed the effects (Fig. 5E). In addition, the ELISA findings demonstrated that TNF-α stimulation markedly elevated the MCP-1, IL-6, and IL-1β levels in adipocytes, which were mitigated by ALM administration (Figs. 5F–H). Then, we detected the macrophage migration assay to evaluate the effects of adipocytes on macrophage recruitment (Fig. 5I); ALM significantly inhibited the macrophages' migration towards adipocytes, while the implications of ALM were counteracted by the co-treatment of 3-TYP (Figs. 5J–K). In addition, we carried out shRNA-mediated Sirt3 knockdown (Sirt3KD) in 3T3-L1 adipocytes. The results consistently demonstrated that genetic inactivation of SIRT3 abolished the beneficial effects of ALM, as shown by a significant reversal of ALM-driven activation of autophagy, and instigated the pro-inflammatory cytokine levels. Specifically, in TNF-α-treated 3T3-L1 adipocytes, SIRT3 knockdown inhibited ALM-driven upregulation of autophagy-related protein levels and restored the production of pro-inflammatory cytokines. (Figs. S3B-G). The above outcomes suggested that ALM mitigates inflammation in adipocytes and reduces the macrophage migration towards adipocytes by activating autophagy mediated by SIRT3.

### ALM nano-emulsion ameliorated ATI through SIRT3-mediated inflammasome suppression

**Fig. 6:**
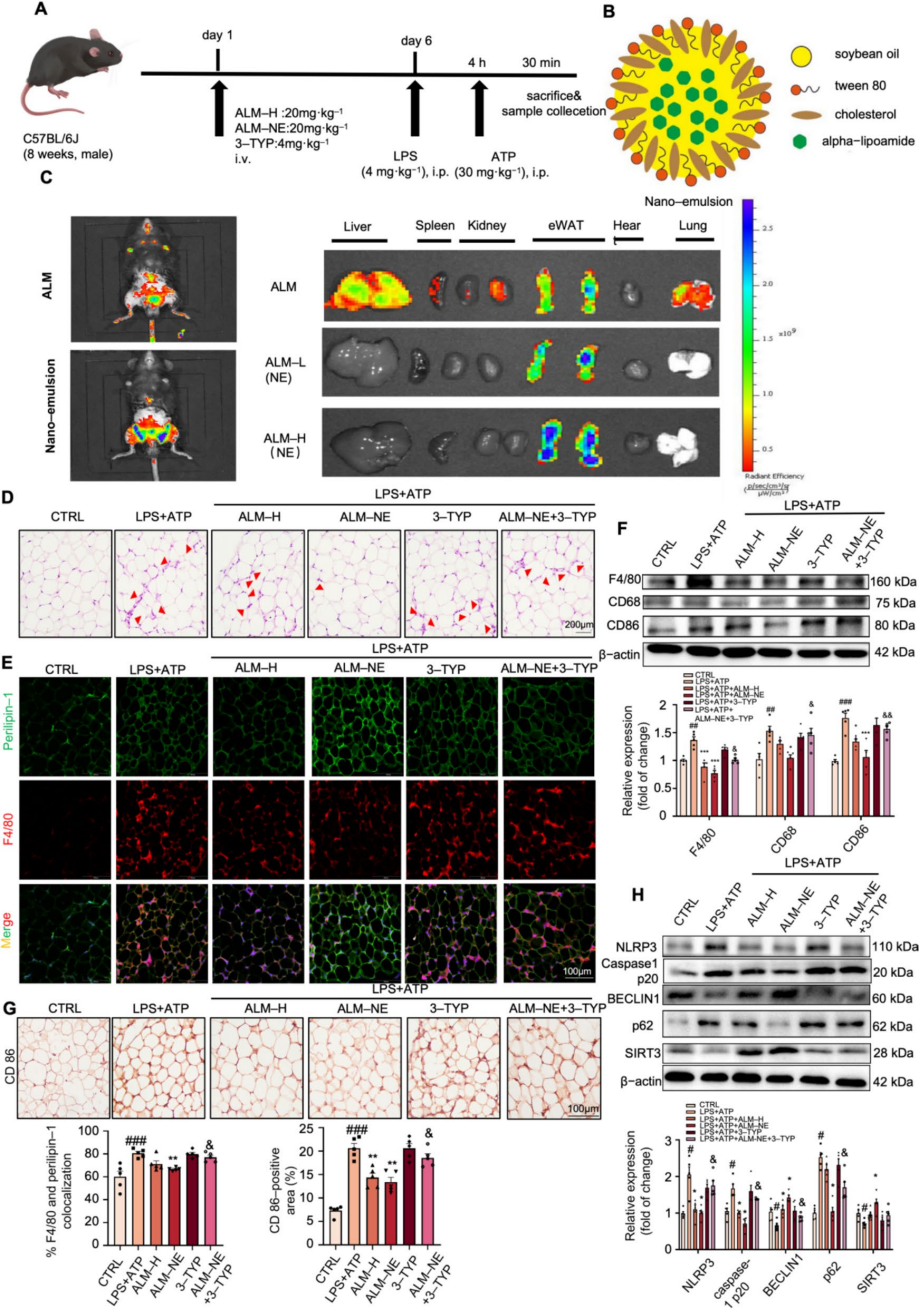
ALM nano-emulsion ameliorated adipose tissue inflammation through SIRT3-mediated inflammasome suppression. **A** The experimental procedure of the LPS plus ATP-stimulated inflammatory mice paradigm. **B** The structure diagram of ALM-nano-emulsion (ALM-NE). **C** Biodistribution of ALM-NE in mice, in vitro imaging of Nile red fluorescence in liver, spleen, kidney, epididymal white adipose tissue (eWAT), heart, and lung (*n* = 5). **D** Representative images of H&E staining of eWAT of adipose tissue from LPS plus ATP-treated mice. (*n* = 5). **E** Immunofluorescence staining of Perilipins (green) and F4/80 (red) in eWAT (*n* = 5). **F** Western blot analysis of F4/80, CD68, and CD86 in adipose tissue from LPS plus ATP-treated mice. (*n* = 4). **G** Immunohistochemical staining of CD86 in eWAT (*n* = 5). **H** Western blot analysis of autophagy-related proteins and NLRP3 and caspase1 p20 in eWAT was analyzed (*n* = 5). Data are expressed as means ± SEM. ^##^*P* < 0.01 and ^###^*P* < 0.001, LPS + ATP vs. CTRL; *P < 0.05, **P < 0.01 and ***P < 0.001, LPS vs ALM-H or ALM-NE; ^&^*P* < 0.05, ^&&^*P* < 0.01, ^&&&^*P* < 0.001, ALM-NE vs. ALM-NE + 3-TYP

In the abovementioned results, we have demonstrated through in vitro experiments that ALM can alleviate the inflammatory response between macrophages and adipocytes. Then, the anti-inflammatory effects of ALM were implemented in LPS and ATP-induced acute inflammation mice (Fig. 6A). Results showed that ALM significantly reduced cytokine secretion in serum and the inflammatory infiltration in adipose tissues (Figs. S4A-C). To further clarify the mitigating effects on adipose tissue inflammation (ATI), we synthesized adipose tissue-targeting ALM nano-emulsion (ALM-NE) (Fig. 6B), which can precisely deliver ALM to adipose tissue, as confirmed by an in vivo imaging system (Fig. 6C). The administration of ALM did not result in apparent toxicity in mice (Figs. S4D-E). The H&E and immunofluorescence staining of the epididymal white adipose tissue (eWAT) section from LPS plus ATP-challenged mice indicated a prominent inflammatory infiltration, which was markedly attenuated by ALM and ALM-NE treatment, with ALM-NE exhibiting superior efficiency (Fig. 6D). Quantitative real-time PCR showed that ALM-NE treatment decreased inflammatory cytokines *Il-6*, *Tnfa*, *Mcp-1*, and *Il-1β* in the eWAT from mice under LPS plus ATP conditions (Figs. S4F–I). And the administration of 3-TYP in combination with ALM resulted in a near-complete reversal of the impact caused by ALM.

Additionally, ALM-NE inhibited the M1 type of macrophages in the eWAT of mice that were subjected to LPS and ATP (Figs. 6E–G). In addition, gene expression analysis demonstrated that ALM treatment effectively mitigated the *F4/80* and *Cd86* mRNA levels in peritoneal macrophages from mice treated with LPS and ATP. However, there was no significant effect on the mRNA level of *Cd206* (Fig. S4J–L). Nevertheless, the concurrent administration of 3-TYP largely counteracted the patterns above.

Furthermore, the administration of ALM-NE resulted in an increase in BECLIN1 and SIRT3 levels while reducing the level of p62 in eWAT. Co-immunoprecipitation analysis revealed that ALM-NE effectively reduced the acetylation level of SOD2 (Fig. S4M). And these effects were reversed when 3-TYP was co-administered (Figs. 6H and S4M). Additionally, ALM-NE consistently hindered the NLRP3 and cleaved caspase-1 levels in the eWAT of mice treated with LPS plus ATP. This effect was reversed by 3-TYP administration simultaneously (Fig. 6H). Taken together, the above outcomes showed that ALM-NE lessened ATI in LPS plus ATP-treated mice by activating SIRT3-mediated autophagy and subsequent inactivation of NLRP3 inflammasome.

### Adipose tissue-targeting ALM-NE alleviated abnormal ATR and obesity in HFD-challenged mice

**Fig. 7:**
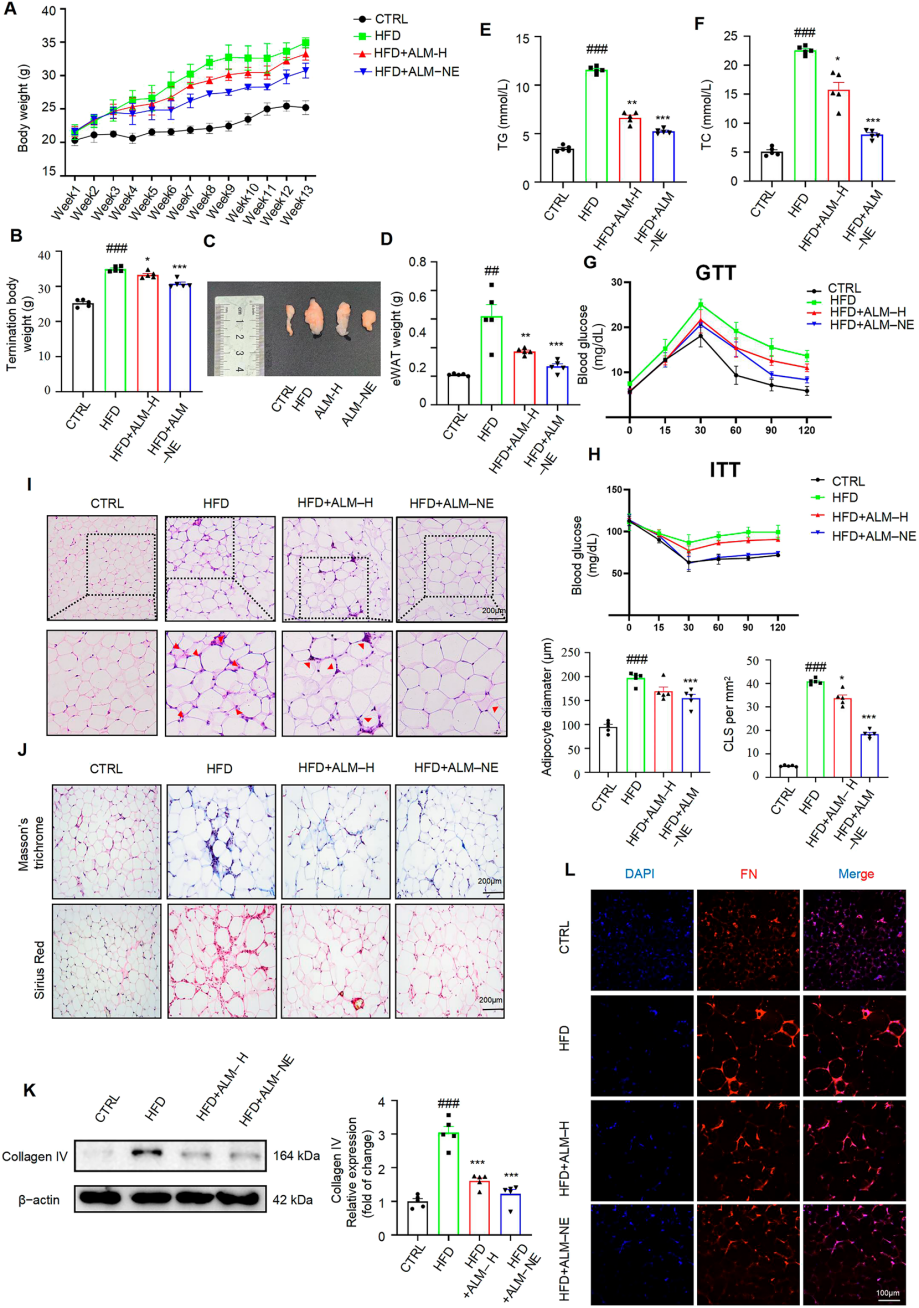
Adipose tissue-targeting ALM-NE alleviated abnormal ATR and obesity in HFD-challenged mice. **A** The body weight of mice in different groups (n = 5). **B** Terminal body weight of mice (*n* = 5). **C** Representative images of eWAT from mice (*n* = 5). **D** Tissue weights of eWAT (*n* = 5). **E**, **F** TG (triglyceride) and TC (total cholesterol) levels of serum in mice (*n* = 5). **G** Intraperitoneal glucose tolerance test (GTT) of varying groups of HFD mice (*n* = 5). **H** Intraperitoneal insulin tolerance test (ITT) of different groups of HFD mice (*n* = 5). **I** Representative images of H&E-stained sections, quantifications of lipid droplets (LDs) diameter, and crown-like structures (CLS) in eWAT from mice (*n* = 5). **J** Representative images of pathological eWAT in mice, as shown by Masson’s trichrome and Sirius red staining (*n* = 5). **K** Western blot analysis of collagen IV in indicated groups of HFD mice (*n* = 5). **L** Immunofluorescence of FN (red) in eWAT of HFD mice. Nuclei were stained with DAPI (blue) (*n* = 5). Data are expressed as means ± SEM (*n* = 5). ^#^*P* < 0.05, HFD vs. CTRL; *P < 0.05, HFD vs ALM-H or ALM-NE; **P < 0.01, HFD vs ALM-H or ALM-NE; ***P < 0.001, HFD vs ALM-H or ALM-NE

Inflammation in the adipose tissue drives abnormal adipose tissue remodeling (ATR) and the progression of obesity. Given the efficiency of ALM-NE in suppressing ATI, we hypothesized that ALM-NE could attenuate abnormal ATR and obesity. Thus, a mouse model of high-fat diet (HFD)-induced obesity was implemented (Fig. S5A). As expected, ALM-NE treatment reversed HFD-induced increase in the body weight (Fig. 7A) and reduced the mass of eWAT (Figs. 7B and C). In addition, the levels of serum lipid parameters, including triglycerides (TG), serum total cholesterol (TC), and low-density lipoprotein cholesterol(LDL-C), were markedly reduced in ALM-NE-treated mice (Figs. 7D–E and S5B) compared to those of HFD-fed mice. ALM-NE improved glucose tolerance and enhanced insulin sensitivity in obese mice (Figs. 7F–G and S5C-E). These findings suggest that adipose tissue-targeting ALM-NE ameliorates adiposity and metabolic dysfunction in HFD-induced obesity.

Obesity-induced abnormal ATR is characterized by macrophage infiltration, extracellular matrix (ECM) overproduction, and adipocyte hypertrophy [28]. Given the beneficial effects of ALM-NE on obesity, we further examined the impact of its influence on abnormal ATR. As shown in Fig. 7H, the histological examination revealed that the adipocyte sizes, dead adipocytes, and macrophage infiltration in eWAT were reduced in ALM-NE treatment. Masson’s trichrome and Sirius red staining showed that collagen deposition and the distinct pattern of collagen distribution in interstitial tissue increased in the eWAT of HFD mice, both of which were attenuated in ALM-NE-treated mice (Figs. 7I and S5F). Consistently, western blotting and immunofluorescent staining analyses indicated that ALM-NE treatment significantly reduced collagen IV and fibronectin (FN) levels in the eWAT of HFD-fed mice (Figs. 7J, K, S5G). Moreover, ALM-NE effectively reduced the elevated levels of IL-1β, TNF-α, IL-6, and MCP-1 in the serum of HFD mice (Figs. S5H-K). These results suggested that ALM ameliorated abnormal ATR in eWAT and consequently induced favorable ECM flexibility, thereby supporting the healthy expansion of adipose tissue.

## Discussion

Abnormal adipose tissue remodeling (ATR), characterized by chronic ATI, ECM deposition, and adipocyte dysfunction, represents a fundamental pathophysiological process in obesity and related metabolic complications.[29]. Within the context, ATI is not merely a symptom but a primary driver of ATR. Pro-inflammatory cytokines released by adipose tissue stimulate the production of ROS, creating oxidative stress. In turn, ROS activate inflammatory signaling pathways, further enhancing the release of inflammatory mediators. Obesity, inflammation, and oxidative stress are intricately linked in a complex interplay that drives the progression of metabolic dysfunction and chronic diseases. Pharmacological approaches targeting pro-inflammatory pathways or oxidative stress are worth exploring for ATR and obesity.

The NLRP3 inflammasome plays a pivotal role in modulating inflammatory responses within dysfunctional adipose tissue and is involved in obesity-related metabolic inflammation. Deficiency of NLRP3 in HFD mice alleviates metabolic disturbances and inflammatory stress [30]. Recent research supports enhanced autophagy, promotes damaged mitochondrial clearance, and inhibits NLRP3 inflammasome activation [31]. The autophagic adaptor, LC3, BECLIN1, SQSTM1/p62, and ULK1 (Unc-51 like autophagy activating kinase 1), involve inactivating NLRP3 inflammasome [32–35]. Autophagy regulates the M1-like macrophage polarization, mitochondrial homeostasis, and inflammasome activation in macrophages [36, 37]. Mitophagy, a cell self-protective mechanism, could alleviate mitochondrial injury. Both mitochondrial injury and mitophagy could be regulated by acetylation/deacetylation of mitochondrial proteins, whereas SIRT3 is the major mitochondrial deacetylase. Here, we demonstrate that ALM-driven SIRT3-mediated mitophagic flux through the mitophagy receptor FUNDC1. Genetic ablation of FUNDC1, abolishing the beneficial effects of ALM, solidifies its indispensability in this cascade, offering a new therapeutic node for intervention. However, we confirmed the ALM enhanced FUNDC1-mediated mitophagy through SIRT3 activation, we did not further interrogate the specific details of structural connections between SIRT3 and FUNDC1, which is worthy of further investigation.

ALM is a promising compound synthesized by combining lipoic acid—a natural antioxidant—with enhanced efficacy [31]. ALM has been shown to outperform lipoic acid in reducing oxidative stress and mitigating mitochondrial dysfunction. However, the molecular target of ALM remains to be fully elucidated. Our present study revealed that ALM enhanced SIRT3 deacetylating activity and increased SIRT3 protein levels in inflammatory models. ALM inhibited NLRP3 inflammasome activation via SIRT3-FUNDC1-mediated mitophagy, thus improving mitochondrial function and eliminating mtROS in RAW264.7 macrophages. Additionally, ALM inhibited the activation of the NLRP3 inflammasome in the eWAT of LPS plus ATP-induced mice by promoting autophagy via the mediation of SIRT3. To validate these findings, we employed autophagy and SIRT3 inhibitors—3-MA and 3-TYP, respectively—which reversed the beneficial effects of ALM on the NLRP3 inflammasome, confirming the involvement of autophagy and SIRT3 in ALM’s mechanism of action. Furthermore, geneic inactivation of SIRT3 abolished the above beneficial effects of ALM, proving compelling genetic evidence for ALM. ALM also proved effective in reducing the inflammatory crosstalk between macrophages and adipocytes, further underscoring its potential therapeutic value. Therefore, ALM suppressed ATI by activating SIRT3-mediated autophagy and suppressing the NLRP3 inflammasome.

Nano-emulsion (NE) refers to homogeneous blends of oil and water phases that are stabilized by surfactant or co-surfactant compounds. NE is suitable to improve solubilization, stability, and bioavailability [38]. Moreover, NE has already been conducted in small molecules that have been verified to possess anti-inflammatory properties, such as resveratrol, etoricoxib, quercetin, piplartine, and astaxanthin [39–43]. Thus, NE was a promising method for improving the bioavailability of small molecules. In the present study, we synthesized an adipose tissue-targeting ALM nano-emulsion (ALM-NE), which can precisely deliver ALM to adipose tissue and showed a better-mitigating impact than dissolved ALM on ATI. This delivery system not only validated the therapeutic potential of targeting adipose tissue SIRT3 in vivo but also dramatically enhanced the efficacy of ALM in alleviating ATI, ATR, and systemic metabolic dysregulation in two distinct mouse models. The efficacy study on ATR showed the importance of the nano-emulsion-based strategy in enhancing the biopharmaceutical properties of ALM.

Taken together, the present study suggests that inhibiting NLRP3 inflammasome activation and enhancing mitochondrial dysfunction through activating autophagy may represent a potential mechanism by which ALM exerts its effects on adipose tissue remodelling, with SIRT3 serving as a promising pharmacological target. ALM ameliorates the inflammatory crosstalk between macrophages and adipocytes and the macrophage infiltration towards adipocytes. In general, we unveil a novel pharmacological strategy for combating obesity-associated adipose tissue dysfunction by delineating a previously unrecognized mechanism through which ALM activates SIRT3 to orchestrate FUNDC1-mediated mitophagy. Collectively, these results position ALM as a promising therapeutic candidate for addressing obesity and its associated metabolic dysfunction, offering a potential pathway to more effective treatments for this widespread and challenging condition.

## Materials and methods

### Reagents and chemicals

**Table:** 

Reagent or resource	Source	Identifier
Fetal bovine serum (FBS)	Bio-channel biotechnology	BC-SE-FBS07
Phosphate-buffered saline (PBS)	Solarbio	P1020
penicillin–streptomycin (P/S)	Solarbio	P1400
trypsin–EDTA	Solarbio	T1300
DAPI	Solarbio	C0065
Lipoamide (ALM)	MedChemExpress	HY-B1142
phorbol 12-myristate 13-acetate (PMA)	MedChemExpress	HY-18739
3-Methyladenine (3-MA)	MedChemExpress	HY-19312
3-TYP	MedChemExpress	HY-108331
3-isobutyl-1-methylxanthine (IBMX)	MedChemExpress	HY-12318
Dexamethasone	MedChemExpress	HY-14648
Adenosine triphosphate (ATP)	Beyotime Biotechnology	ST1092
mRFP-GFP-LC3	Beyotime Biotechnology	C3011
RIPA lysis buffer	Beyotime Biotechnology	P0013C
Lipofectamine 3000	ThermoFisher Scientific	L3000150
BCA protein assay kit	ThermoFisher Scientific	23225
DMEM medium	ThermoFisher Scientific	#21010046
TRIzol reagent	ThermoFisher Scientific	#15596026
3-(4,5-dimethylthiazol-2-yl)- 2,5-diphenyltetrazolium bromide (MTT)	Bioss	C-0068
Lipopolysaccharides (LPS)	Sigma-Aldrich	L4391
Griess reagent	Sigma-Aldrich	G4410
Nile Red	Sigma-Aldrich	72485
Mouse IL-6 ELISA kit	Neobioscience Technology	EMC004
Mouse IL-1β ELISA kit	Neobioscience Technology	EMC001b
Mouse TNF-α ELISA kit	Neobioscience Technology	EMC102a
Mouse MCP-1 ELISA kit	Neobioscience Technology	EMC113
Mouse IL-18 ELISA kit	Neobioscience Technology	EMC011
FUNDC1 siRNA	Santa Cruz	SC-145273

### Cell culture and treatment

Mouse RAW264.7 macrophages were acquired from ATCC (Manassas, VA, USA) and grown in DMEM with 10% FBS and 1% P/S at 37 ℃. The 3T3-L1 mouse preadipocytes were acquired from ATCC and cultured in DMEM supplemented with 10% FBS and 1% P/S. The RAW264.7 macrophages were treated beforehand either with ALM (25 μg ml^−1^) and LPS (1 μg ml^−1^) for a duration of 18 h. Afterward, the cells were exposed to treatment with other indicated inhibitors for 6 h and then treated with 1 mM ATP for 1 h. 3T3-L1 preadipocytes underwent differentiation into fully developed adipocytes, as previously shown [44, 45]. Shortly after reaching confluence 2 days before the experiment started (day 0), the cells were exposed to a differentiation medium. The experimental medium comprised 0.5 mM 3-Isobutyl-1-Methylxanthine (IBMX), 1 μM dexamethasone (DEX), and 5 μg ml^−1^ insulin. The differentiation medium was prepared by supplementing DMEM with 10% FBS. On the second day, the cells were transferred to DMEM enriched with 10% FBS and 5 μg ml^−1^ insulin for 6 days. A daily replacement of the medium occurred. The cells were incubated at 37 ℃ in a humidified incubator with 5% CO_2_ for further examination.

### Cell viability assay

The RAW264.7 cells viability was ascertained by employing the 3-(4,5-dimethylthiazol-2-yl)-2,5-diphenyltetrazolium bromide (MTT) test, following a previously established method [46, 47]. In summary, cells were evenly allocated onto 96-well plates, with a concentration of 1 × 10^4^ cells per well. Before usage, the ALM administration was diluted in DMSO (10 mg ml^−1^) with DMEM complete medium. Next, the cell viability was assessed by incubating with DMEM solution containing MTT (1 mg ml^−1^) for 30 min. Subsequently, 100 μL of DMSO was added to each well to dissolve the formazan precipitates. The microplate reader (Thermo Fisher Scientific, Waltham, MA, USA) was used to measure the absorbance at 570 nm. Cell Counting Kit 8 (CCK8) reagent (Beyotime, China) was used to determine the cell viability of the indicated concentration of ALM. Incubation took place at 37 °C for 2 h, and the plate was analyzed with a microplate reader at 450 nm in order to measure absorbance. Cell viability was assessed by quantifying it as a percentage compared to that of the control cells. Cell viability = (experimental OD value—NC OD value/Control OD value—NC OD value) × 100%

### Nitric oxide assay

As described previously, nitric oxide was measured using a commercial Griess reagent to determine total nitrate [48]. Briefly, RAW264.7 cells were seeded (1 × 10^4^ per well) in 96-well plates following LPS treatment for the indicated concentration; then, the supernatant was gathered and combined with an equal amount of Griess reagent on a fresh 96-well plate. After being incubated at room temperature (RT) for 15 min, the absorbance at 540 nm was quantified employing a microplate reader (Thermo Fisher Scientific, Waltham, MA, USA). A sodium nitrite standard curve assessed the nitrate concentration.

### Enzyme-linked immunosorbent assay (ELISA)

The IL-1β, IL-6, MCP-1, IL-18, and TNF-α cytokine levels in both cell culture media and serum were measured using an ELISA kit (Neobioscience Technology Co., Ltd., Shenzhen, China), adhering to the directions provided by the manufacturer [49]. The cell medium and plasma obtained from mice were centrifuged at a force of 1500 g for 10 min at 4 ℃. The supernatant was preserved at – 20 ℃ for further analysis.

### Western blotting (WB)

WB technique was executed using the previously published protocol [50]. RAW264.7 macrophages, 3T3-L1 adipocytes, or epididymal adipose tissue from mice were broken down employing RIPA lysis buffer. The quantification of protein content was performed with a BCA Protein Assay kit. Protein samples containing 10–20 μg were separated using SDS-PAGE and then placed on PVDF membranes (PALL, USA). Subsequently, the membranes were obstructed by deploying a solution of 5% nonfat milk in TBST buffer (100 mmol L^−1^ of NaCl, 10 mmol L^−1^ of Tris–HCl, pH 7.5, and 0.1% Tween 20) for a period of 1 h at RT. The proteins were probed overnight at 4℃ using specific primary antibodies. Following three washes with TBST, the appropriate secondary antibodies were applied and incubated at RT for 1 h. The protein bands were visualized using an enzyme-linked chemiluminescence reagent using the Tanon image system (Tanon, Shanghai, China) and quantified using Image J (NIH, USA). The antibodies we used were listed as follows:

**Table:** 

Antibody	Catalog	Company	Application	Dilution
SIRT3	5490S	Cell Signaling Technology Danvers, MA	WB	1:1000
SIRT1	13161-1-AP	Proteintech, China	WB	1:1000
SIRT6	13572-1-AP	Proteintech, China	WB	1:1000
SIRT4	IPB9097	Baijia, China	WB	1:1000
FUNDC1	28519-1-AP	Proteintech, China	WB	1:1000
PARK2/Parkin	14060-1-AP	Proteintech, China	WB	1:1000
Fibronectin	15613-1-AP	Proteintech, China	IF	1:200
Collagen Type IV	19674-1-AP	Proteintech, China	WB	1:1000
NLRP3	68102-1-Ig	Proteintech, China	WB/IF	1:5000/1:200
ASC	67494-1-Ig	Proteintech, China	IF	1:200
CASPASE-1	22915-1-AP	Proteintech, China	WB/IF	1:1000/1:200
IL-1β	16806-1-AP	Proteintech, China	WB/IF	1:1000/1:200
NF-κB p65 Polyclonal antibody	10745-1-AP	Proteintech, China	WB	1:1000
Phospho-NF-κB p65 (Ser536)	3033	Cell Signaling Technology Danvers, MA	WB	1:1000
SQSTM1	23214S	Cell Signaling Technology Danvers, MA	WB/IP	1:1000/1:20
ATG5	12994 T	Cell Signaling Technology Danvers, MA	WB	1:1000
LC3	12741S	Cell Signaling Technology Danvers, MA	WB	1:1000
BECN1	3495S	Cell Signaling Technology Danvers, MA	WB	1:1000
SIRT2	SC-28298	Santa Cruz Biotechnology (Santa Cruz, CA, USA)	WB	1:500
SIRT5	SC-271635	Santa Cruz Biotechnology (Santa Cruz, CA, USA)	WB	1:500
CD86	SC-19617	Santa Cruz Biotechnology (Santa Cruz, CA, USA)	IHC	1:100
Perilipin-1	9349S	Cell Signaling Technology Danvers, MA	IF	1:200
F4/80	SC-377009	Santa Cruz Biotechnology (Santa Cruz, CA, USA)	IF	1:200
Mouse polyclonal anti-ACTB/β-actin	SC-20060	Santa Cruz Biotechnology (Santa Cruz, CA, USA)	WB	1:1000
Mouse Anti-PINK1 antibody	bsm-51265M	Bioss Woburn, MA, USA	WB	1:1000
Goat Anti-Rabbit IgG H&L/Alexa fluor 488	Bs-0295G-AF488	Bioss Woburn, MA, USA	IF	1:500
Goat Anti-Mouse IgG H&L/Alexa fluor 555	bs-0296G-AF555	Bioss Woburn, MA, USA	IF	1:500
Anti-mouse	211130	Biopm Wuhan, China	WB	1:10000
Anti-Rabbit	220362	Biopm Wuhan, China	WB	1:8000

### Quantitative real-time polymerase chain reaction (qRT-PCR)

Total RNA was extracted by deploying TRIzol reagent (Invitrogen, Carlsbad, CA, USA) following the manufacturer's directions. The bands were converted into cDNA, deploying the Prime Script RT master mix. The qRT-PCR analysis was performed using the SYBR Green Master Mix and gene-specific primers. The fold change was normalized by *Gapdh* and determined using the 2^−ΔΔCt^ technique. The oligonucleotide primer pairs used in qRT-PCR were listed as follows:

**Table:** 

Gene	Forward	Reverse
Gapdh	CATTTCACTCAAGGTTGTC	ATCATACTTGGCAGGTTTCTCC
Cd86	ATCTGCCGTGCCCATTTACA	CAACTTTTGCTGGTCCTGCC
F4/80	CTTTGGCTATGGGCTTCCAGTC	GCAAGGAGGACAGAGTTTATCGTG
Cd206	CAGGTGTGGGCTCAGGTAGT	TGTGGTGAGCTGAAAGGTGA
IL-1β	TGTTCTTTGAAGTTGACGGACCC	TCATCTCGGAGCCTGTAGTGC
TNF-α	GAGAAAGTCAACCTCCTCTCTG	GAAGACTCCTCCCAGGTATATG
Mcp-1	CAACTCTCACTGAAGCCAGCTC	TAGCTCTCCAGCCTACTCATTGG
Il-6	CCAGAGATACAAAGAAATGATGG	ACTCCAGAAGACCAGAGGAAAT
Sirt3	TCACAACCCCAAGCCCTTTT	TATAGAGCCGCAGAAGCAGC

### Immunofluorescence staining

Immunofluorescence staining was conducted according to the previously reported method [51]. In brief, the cell coverslips and epididymal adipose tissue slices were treated with 4% paraformaldehyde for 10 min to fix them. Subsequently, they were incubated in a solution containing 0.5% Triton X-100 for 10 min to allow permeabilization of the cytomembrane. Next, the slides were treated with a 1% BSA solution in PBS for 1 h, followed by overnight incubation at 4℃ with various primary antibodies. The primary antibodies used were as follows: Fibronectin (1:200, proteintech); IL-1β (1:200, proteintech); NLRP3 (1:200, proteintech); Caspase-1 (1:200, proteintech); ASC (1:200, proteintech); Perilipin-1 (1:200, Cell Signaling Technology); F4/80 (1:100, Santa Cruz Biotechnology). Afterward, cells or epididymal adipose tissues were rinsed with PBS three times and then exposed to the appropriate Alexa 488 or 555 conjugated antibody for 2 h at RT. The nuclei were labeled with 4',6-diamidino-2-phenylindole (DAPI). Observations were performed on an FV3000 laser-scanning confocal microscope system (Olympus).

### Determination of mitochondrial membrane potential (MMP)

The JC-1 kit (Yeasen, Shanghai, China) was deployed to conduct an MMP experiment. The RAW264.7 macrophages were placed in a solution containing 1 μg ml^−1^ of JC-1 and cultured for 20 min at 37 ℃. Following two rinses with PBS, the confocal laser scanning microscope measured the fluorescence intensity of the dispersed JC-1 monomers and aggregates. The monomers emit a green fluorescence, while the aggregates emit a red fluorescence.

### Measurements of intracellular ROS, mitochondrial ROS and SIRT3 activity assay

The ROS Assay Kit (S0033, Beyotime, China) measured the level of ROS in RAW264.7 macrophages. The amounts of superoxide in the macrophages were quantified using MitoSOX Red (Yeasen, Shanghai, China). The cells were washed twice with PBS and then exposed to a 5 μM concentration of MitoSOX for 10 min. Afterward, the cells were rinsed twice with PBS, and the cellular fluorescence intensity was quantified by flow cytometry.

For the measurement of SIRT3 activity, RAW264.7 macrophages treated with or without ALM and 3-TYP were lysed with RIPA lysis buffer. SIRT3 activity was determined by a commercial assay kit (Epigentek, P-4037-48) according to the manufacturer’s instructions. Fluorometric intensities were measured with a microplate fluorometer (excitation wavelength = 530 nm, emission wavelength = 590 nm).

### Macrophages migration assay

The macrophages migration assay was performed as described previously [52]. In summary, fully differentiated 3T3-L1 adipocytes were incubated in the presence or absence of ALM (25 μg·ml^−1^) and TNF-α (15 ng·ml^−1^) for a period of 24 h. Subsequently, the adipocytes were treated with 3-TYP for 6 h. At a density of 5 × 10^4^ cells per well, the inserts were seeded with RAW264.7 macrophages. After gathering the conditional medium from 3T3-L1 cells, it was transferred to 24-well plates containing inserts. Following a 4 h migration period at 37 ℃, the macrophages in the bottom compartment were immobilized using a 4% formaldehyde solution for 20 min. Subsequently, the macrophages were stained with DAPI, and their quantification was performed using Image J software.

### Molecular docking

The molecular docking process employed AutoDock 4.2 (The Scripps Research Institute, La Jolla, CA, USA). The receptor employed was the crystal structure of the quaternary complex, namely the complex between SIRTs family and the agonist Amiodarone hydrochloride: SIRT1 (PDB ID: 4ZZH), SIRT2 (PDB ID: 1J8F), SIRT3 (PDB ID: 5H4D), SIRT4 (PDB ID: 5OJN), SIRT5 (PDB ID: 8GBL), SIRT6 (PDB ID: 5X16), SIRT7 (PDB ID: 5IQZ). The protein was first produced at a pH of 7.4 by removing all water molecules and adding the appropriate hydrogen atoms. The 3D configuration of ALM was obtained from the PubChem database. The Gasteiger charge was assigned, and a grid box of 50 Å × 50 Å × 50 Å with a spacing of 500 Å was placed to encompass the catalytic cleft surface, with the assistance of amiodarone hydrochloride. The genetic algorithm was selected for conducting docking calculations, and 50 iterations of the genetic algorithm were executed. The obtained postures were grouped with a tolerance of 2.0 Å. The PyMOL program was used to depict the molecular docking with the lowest binding energy.

### Cellular thermal shift assay (CETSA)

The CETSA experiment was conducted using the previously disclosed methodology [53]. In summary, RAW264.7 cells were pretreated with or without 25 μg·ml^−1^ ALM for 18 h. The cells were disrupted using RIPA lysis buffer and cooled on ice for 10 min. Subsequently, they underwent centrifugation at 12,000 g for 10 min at 4℃. The liquid portion was collected, and then the BCA Protein Assay kit was deployed to calibrate the protein content to 1 μg·μL^−1^. The solutions were heated at the specified temperatures (50–75 ℃) for 5 min using a thermal cycler. The soluble supernatant was submitted to WB after being centrifuged for 20 min at 12,000 g at a temperature of 4 ℃.

### Co-immunoprecipitation

The RAW264.7 cell lysates were subjected to overnight incubation with the specified antibodies on a rotator at a temperature of 4 ℃. The primary antibodies were incubated with primary antibody (NLRP3, 1:50, Proteintech); SOD2, 1:50, Proteintech; Acetylated-Lysine, 1:50, Cell Signaling Technology). Next, 20 μL of protein A/G-agarose beads (Santa Cruz Biotechnology) were added and incubated on a rotator for 2 h at RT. Subsequently, the precipitates underwent three rounds of cold PBS washing. The bound proteins were reconstituted and heated in 2× loading buffer before being used for WB investigation.

### Histological analysis

For histological analysis, epididymal adipose tissues (eWAT) were collected and fixed in 4% paraformaldehyde. Then, 8 μm sections cut from paraffin tissue slides were stained with hematoxylin and eosin (H&E), Masson’s trichrome, and Sirius Red, depending on standard protocols, and observed under an optical microscope (Olympus, Japan).

### Immunohistochemistry

The epididymal adipose tissue Sects. (8 μm) were prepared, and the slices were rinsed in PBS three times, followed by treatment with 3% H_2_O_2_ to inhibit endogenous peroxidase activity for 10 min. Following this, normal goat serum was employed to minimize non-specific staining for 1 h at RT. Subsequently, the slides were incubated overnight with the primary antibodies in a PBS solution containing 5% BSA at a temperature of 4 ℃. The primary antibodies used were CD86 (1:100, Santa Cruz Biotechnology). The slides were rinsed and exposed to secondary antibodies for 30 min at RT, followed by diaminobenzidin (DAB) for 10 min. The nucleus was stained with hematoxylin. Observations were performed on an optical microscope (Olympus, Japan).

### Preparation of adipose tissues-targeting ALM nano-emulsion

The ALM nano-emulsion (ALM-NE) was prepared based on a self-emulsifying method. ALM (50 mg), soybean oil (0.2 g; Sigma, S7381), Tween-80 (0.6 g; MCE, HYY1891), glycerin (0.7 g; Sigma, PHR1020), and cholesterol (0.7 mg; Sigma, C8667) were mixed and vortexed. Then, add ultrapure water to 25 ml. Use a high-pressure homogenizer to homogenize at low temperatures for 10 min. Thus, ALM-NE (2 mg ml^−1^) was obtained. The particle size of the prepared nanoparticles (ALM-NE) was determined by a dynamic light scattering (DLS) analyzer (Brookhaven Instruments Co., Ltd, NY, USA). In vivo, the biodistribution of ALM-NE was performed qualitatively. The mice were randomly divided into four groups (n = 5). 200 μL ALM-NE were labeled with Nile Red (0.1 mg ml^−1^) and delivered into mice via tail injection for 1 h. The control mice were similarly infused with free Nile Red. Then, animals were euthanized, and organs were harvested. In vivo and ex vivo fluorescent images of mice were acquired using a small animal imaging system (PerkinElmer Inc., Waltham, Massachusetts, USA).

### LPS plus ATP-induced acute inflammation mice model

Forty-five C57BL/6 mice were allocated into nine groups based on their body weight (*n* = 5): The control (40% PEG 400 solution), model (40% PEG 400 solution), DEX (0.5 mg·ml^−1^ dexamethasone dissolved in 40% PEG 400), ALM-L (0.5 mg·ml^−1^ ALM dissolved in 40% PEG 400), ALM-M (1 mg·ml^−1^ ALM dissolved in 40% PEG400), ALM-H (2 mg·ml^−1^ ALM dissolved in 40% PEG400), ALM-NE (2 mg·ml^−1^ ALM-NE), 3-TYP (0.4 mg·ml^−1^ 3-TYP dissolved in 40% PEG 400), 3-TYP (0.4 mg·ml^−1^ 3-TYP dissolved in 40% PEG 400) + ALM-NE (2 mg·ml^−1^ ALM-NE). The mice in each group received intravenous injections of 200 μL indicated solution daily for 5 days. On the sixth day, the control mice were intraperitoneally given an injection of PBS, and the remaining groups of mice received an intraperitoneal infusion of 4 mg·kg^−1^ LPS for 4 h, then an intraperitoneal injection of 30 mg·kg^−1^ of ATP for 30 min. Subsequently, blood specimens were obtained from mice under the influence of isoflurane anesthesia. The epididymal adipose tissues were gathered and preserved at -80℃.

### High-fat diet-induced obesity mice model

Five groups of male C57BL/6 mice weighing 20–25 g were fed a high-fat diet (HFD, 60% fat in calories; Dyets No. 112252, Bethlehem, PA) or its respective regular chow (Control) diet for 13 weeks. From the third week, the control and HFD mice were intravenously administered 200 μL of 40% PEG 400 solution; the other two groups of mice received intravenous injections of the same amount of ALM-H (2 mg ml^−1^ ALM dissolved in 40% PEG400) and ALM-NE (2 mg ml^−1^ ALM-NE) for 11 weeks. The glucose tolerance test (GTT) in vivo was conducted using methods previously described [51]. Mice were fasted overnight, and then blood was collected from the mice's tails and tested with a commercial glucometer (Yuyue, 580). Mice were orally treated with glucose solutions (2 g/kg) after 12 h fasting. For the insulin tolerance test (ITT), mice were fed ad libitum and administered insulin (0.75 units/kg body weight; Tong Hua Dong Bao Group, S20020092). Then, the blood glucose levels of the mice were measured at 0, 30, 60, 90, and 120 min. At the end of the treatment, mice were euthanized to harvest serum and epididymal adipose tissues.

### Statistical analysis

The statistical analysis included presenting all data as means ± SEM, calculated based on a minimum of three separate trials. The study used Graphpad 8 software (Graphpad Software, San Diego, CA, USA). A one-way ANOVA with Bonferroni's multiple-comparisons post hoc test was used to compare more than two groups. A two-tailed Student's t-test was deployed to compare the two groups. Significance was ascertained at a p-value of less than 0.05.

## Supplementary Information

Supplementary Material 1.

## Data Availability

All data generated or analysed during this study are included in this published article and its supplementary information files.

## Abbreviations

3-MA: 3-Methyladenine
ATI: Adipose tissue inflammation
ALM: Alpha lipoamide
ATP: Adenosine triphosphate
ATR: Adipose tissue remodeling
CM: Conditional medium
eWAT: Epididymal white adipose tissue
HFD: High-fat diet
LA: Lipoic acid
LC3: Microtubule-associated protein light chain 3
IL-1β: Interleukin-1β
LPS: Lipopolysaccharides
MCP-1: Monocyte chemoattractant protein 1
MMP: Mitochondrial membrane potential
mtROS: Mitochondrial reactive oxygen species
NE: Nano-emulsion
NLRP3: NOD-like receptor thermal protein domain associated protein 3
NO: Nitric oxide
SOD2: Mitochondrial superoxide dismutase
TNF-α: Tumor necrosis factor-α

## References

[1] Koenen M, et al. Obesity, adipose tissue and vascular dysfunction. Circ Res. 2021;128(7):951–68. 33793327 10.1161/CIRCRESAHA.121.318093 PMC8026272

[2] Vishvanath L, Gupta RK. Contribution of adipogenesis to healthy adipose tissue expansion in obesity. J Clin Invest. 2019;129(10):4022–31. 31573549 10.1172/JCI129191 PMC6763245

[3] Li H, et al. Macrophages, chronic inflammation, and insulin resistance. Cells. 2022. 10.3390/cells11193001. 36230963 10.3390/cells11193001 PMC9562180

[4] Latz E, Xiao TS, Stutz A. Activation and regulation of the inflammasomes. Nat Rev Immunol. 2013;13(6):397–411. 23702978 10.1038/nri3452 PMC3807999

[5] Zou J, et al. Ginsenoside Rk2, a dehydroprotopanaxadiol saponin, alleviates alcoholic liver disease via regulating NLRP3 and NLRP6 inflammasome signaling pathways in mice. J Pharm Anal. 2023;13(9):999–1012. 37842661 10.1016/j.jpha.2023.05.005 PMC10568107

[6] Nakahira K, et al. Autophagy proteins regulate innate immune responses by inhibiting the release of mitochondrial DNA mediated by the NALP3 inflammasome. Nat Immunol. 2011;12(3):222–30. 21151103 10.1038/ni.1980 PMC3079381

[7] Li R, et al. Therapeutic effect of Sirtuin 3 on ameliorating nonalcoholic fatty liver disease: the role of the ERK-CREB pathway and Bnip3-mediated mitophagy. Redox Biol. 2018;18:229–43. 30056271 10.1016/j.redox.2018.07.011 PMC6079484

[8] Qin Y, et al. Impaired autophagy in microglia aggravates dopaminergic neurodegeneration by regulating NLRP3 inflammasome activation in experimental models of Parkinson’s disease. Brain Behav Immun. 2021;91:324–38. 33039664 10.1016/j.bbi.2020.10.010

[9] Jessop F, et al. Autophagy deficiency in macrophages enhances NLRP3 inflammasome activity and chronic lung disease following silica exposure. Toxicol Appl Pharmacol. 2016;309:101–10. 27594529 10.1016/j.taap.2016.08.029 PMC5054752

[10] Kim SH, et al. Ezetimibe ameliorates steatohepatitis via AMP activated protein kinase-TFEB-mediated activation of autophagy and NLRP3 inflammasome inhibition. Autophagy. 2017;13(10):1767–81. 28933629 10.1080/15548627.2017.1356977 PMC5640190

[11] Bai D, et al. ALDOA maintains NLRP3 inflammasome activation by controlling AMPK activation. Autophagy. 2022;18(7):1673–93. 34821530 10.1080/15548627.2021.1997051 PMC9298449

[12] Hirschey MD, et al. Sirt3 regulates mitochondrial fatty-acid oxidation by reversible enzyme deacetylation. Nature. 2010;464(7285):121–5. 20203611 10.1038/nature08778 PMC2841477

[13] Liu F, et al. S-sulfhydration of SIRT3 combats BMSC senescence and ameliorates osteoporosis via stabilizing heterochromatic and mitochondrial homeostasis. Pharmacol Res. 2023;192:106788. 37146925 10.1016/j.phrs.2023.106788

[14] Li M, et al. Genistein mitigates senescence of bone marrow mesenchymal stem cells via ERRα-mediated mitochondrial biogenesis and mitophagy in ovariectomized rats. Redox Biol. 2023;61:102649. 36871183 10.1016/j.redox.2023.102649 PMC9995482

[15] Li X, et al. Targeting SIRT3 sensitizes glioblastoma to ferroptosis by promoting mitophagy and inhibiting SLC7A11. Cell Death Dis. 2024;15(2):168. 38395990 10.1038/s41419-024-06558-0 PMC10891132

[16] Tao R, et al. Regulation of MnSOD enzymatic activity by Sirt3 connects the mitochondrial acetylome signaling networks to aging and carcinogenesis. Antioxid Redox Signal. 2014;20(10):1646–54. 23886445 10.1089/ars.2013.5482 PMC3942696

[17] Yu W, et al. Dexmedetomidine ameliorates hippocampus injury and cognitive dysfunction induced by hepatic ischemia/reperfusion by activating SIRT3-mediated mitophagy and inhibiting activation of the NLRP3 inflammasome in young rats. Oxid Med Cell Longev. 2020;2020:7385458. 34493950 10.1155/2020/7385458 PMC8418694

[18] Bisby RH, Parker AW. Antioxidant reactions of dihydrolipoic acid and lipoamide with triplet duroquinone. Biochem Biophys Res Commun. 1998;244(1):263–7. 9514912 10.1006/bbrc.1998.8245

[19] Li X, et al. Lipoamide protects retinal pigment epithelial cells from oxidative stress and mitochondrial dysfunction. Free Radic Biol Med. 2008;44(7):1465–74. 18258206 10.1016/j.freeradbiomed.2008.01.004 PMC2597696

[20] Zhang HF, et al. Alpha lipoamide inhibits diabetic kidney fibrosis via improving mitochondrial function and regulating RXRα expression and activation. Acta Pharmacol Sin. 2023;44(5):1051–65. 36347997 10.1038/s41401-022-00997-1 PMC10104876

[21] Zhou B, et al. Alpha lipoamide ameliorates motor deficits and mitochondrial dynamics in the Parkinson’s disease model induced by 6-hydroxydopamine. Neurotox Res. 2018;33(4):759–67. 29019159 10.1007/s12640-017-9819-5

[22] Zhao L, et al. Lipoamide acts as an indirect antioxidant by simultaneously stimulating mitochondrial biogenesis and phase II antioxidant enzyme systems in ARPE-19 cells. PLoS ONE. 2015;10(6):e0128502. 26030919 10.1371/journal.pone.0128502 PMC4452644

[23] Karam BS, et al. Oxidative stress and inflammation as central mediators of atrial fibrillation in obesity and diabetes. Cardiovasc Diabetol. 2017;16(1):120. 28962617 10.1186/s12933-017-0604-9 PMC5622555

[24] Kelley N, et al. The NLRP3 inflammasome: an overview of mechanisms of activation and regulation. Int J Mol Sci. 2019. 10.3390/ijms20133328. 31284572 10.3390/ijms20133328 PMC6651423

[25] Liu P, et al. Sirtuin 3-induced macrophage autophagy in regulating NLRP3 inflammasome activation. Biochim Biophys Acta (BBA). 2018;1864(3):764–77. 10.1016/j.bbadis.2017.12.027 29277324

[26] Zhou R, et al. Thioredoxin-interacting protein links oxidative stress to inflammasome activation. Nat Immunol. 2010;11(2):136–40. 20023662 10.1038/ni.1831

[27] Zhou X, et al. Resveratrol regulates mitochondrial reactive oxygen species homeostasis through Sirt3 signaling pathway in human vascular endothelial cells. Cell Death Dis. 2014;5(12):e1576. 25522270 10.1038/cddis.2014.530 PMC4454164

[28] Unamuno X, et al. Nlrp3 inflammasome blockade reduces adipose tissue inflammation and extracellular matrix remodeling. Cell Mol Immunol. 2021;18(4):1045–57. 31551515 10.1038/s41423-019-0296-z PMC8115140

[29] Sun K, Kusminski CM, Scherer PE. Adipose tissue remodeling and obesity. J Clin Invest. 2011;121(6):2094–101. 21633177 10.1172/JCI45887 PMC3104761

[30] Sokolova M, et al. Nlrp3 inflammasome deficiency attenuates metabolic disturbances involving alterations in the gut microbial profile in mice exposed to high fat diet. Sci Rep. 2020;10(1):21006. 33273482 10.1038/s41598-020-76497-1 PMC7712828

[31] Ding Z, et al. LOX-1, mtDNA damage, and NLRP3 inflammasome activation in macrophages: implications in atherogenesis. Cardiovasc Res. 2014;103(4):619–28. 24776598 10.1093/cvr/cvu114 PMC4200051

[32] Su SH, et al. Urb597 protects against NLRP3 inflammasome activation by inhibiting autophagy dysfunction in a rat model of chronic cerebral hypoperfusion. J Neuroinflammation. 2019;16(1):260. 31815636 10.1186/s12974-019-1668-0 PMC6900848

[33] Dagvadorj J, et al. Recruitment of pro-IL-1α to mitochondrial cardiolipin, via shared LC3 binding domain, inhibits mitophagy and drives maximal NLRP3 activation. Proc Natl Acad Sci U S A. 2021. 10.1073/pnas.2015632118. 33361152 10.1073/pnas.2015632118 PMC7817159

[34] Houtman J, et al. Beclin1-driven autophagy modulates the inflammatory response of microglia via NLRP3. EMBO J. 2019. 10.15252/embj.201899430. 30617086 10.15252/embj.201899430 PMC6376276

[35] Kimura T, et al. Trim-directed selective autophagy regulates immune activation. Autophagy. 2017;13(5):989–90. 26983397 10.1080/15548627.2016.1154254 PMC5446080

[36] Liu K, et al. Impaired macrophage autophagy increases the immune response in obese mice by promoting proinflammatory macrophage polarization. Autophagy. 2015;11(2):271–84. 25650776 10.1080/15548627.2015.1009787 PMC4502775

[37] Kim MJ, et al. SESN2/sestrin2 suppresses sepsis by inducing mitophagy and inhibiting NLRP3 activation in macrophages. Autophagy. 2016;12(8):1272–91. 27337507 10.1080/15548627.2016.1183081 PMC4968237

[38] Chen BH, Inbaraj BS. Nanoemulsion and nanoliposome based strategies for improving anthocyanin stability and bioavailability. Nutrients. 2019. 10.3390/nu11051052. 31083417 10.3390/nu11051052 PMC6566753

[39] Clark MA, Jepson MA, Hirst BH. Exploiting m cells for drug and vaccine delivery. Adv Drug Deliv Rev. 2001;50(1–2):81–106. 11489335 10.1016/s0169-409x(01)00149-1

[40] Md S, et al. Development and evaluation of repurposed Etoricoxib loaded nanoemulsion for improving anticancer activities against lung cancer cells. Int J Mol Sci. 2021. 10.3390/ijms222413284. 34948081 10.3390/ijms222413284 PMC8705699

[41] Samadi A, et al. Ameliorating quercetin constraints in cancer therapy with pH-responsive agarose-polyvinylpyrrolidone -hydroxyapatite nanocomposite encapsulated in double nanoemulsion. Int J Biol Macromol. 2021;182:11–25. 33775763 10.1016/j.ijbiomac.2021.03.146

[42] Fofaria NM, et al. Nanoemulsion formulations for anti-cancer agent piplartine–characterization, toxicological, pharmacokinetics and efficacy studies. Int J Pharm. 2016;498(1–2):12–22. 26642946 10.1016/j.ijpharm.2015.11.045 PMC4718800

[43] Haung HY, et al. A novel oral Astaxanthin nanoemulsion from Haematococcus pluvialis induces apoptosis in lung metastatic melanoma. Oxid Med Cell Longev. 2020;2020:2647670. 32908627 10.1155/2020/2647670 PMC7471791

[44] Granata R, et al. Obestatin regulates adipocyte function and protects against diet-induced insulin resistance and inflammation. FASEB J. 2012;26(8):3393–411. 22601779 10.1096/fj.11-201343

[45] Zhang T, et al. SIRT3 acts as a positive autophagy regulator to promote lipid mobilization in adipocytes via activating AMPK. Int J Mol Sci. 2020. 10.3390/ijms21020372. 31936019 10.3390/ijms21020372 PMC7013837

[46] Zhang T, et al. Sirt3 promotes lipophagy and chaperon-mediated autophagy to protect hepatocytes against lipotoxicity. Cell Death Differ. 2020;27(1):329–44. 31160717 10.1038/s41418-019-0356-z PMC7206074

[47] Linghu KG, et al. Small molecule deoxynyboquinone triggers alkylation and ubiquitination of Keap1 at Cys489 on Kelch domain for Nrf2 activation and inflammatory therapy. J Pharm Anal. 2024;14(3):401–15. 38618249 10.1016/j.jpha.2023.07.009 PMC11010449

[48] Goshi E, Zhou G, He Q. Nitric oxide detection methods in vitro and in vivo. Med Gas Res. 2019;9(4):192–207. 31898604 10.4103/2045-9912.273957 PMC7802420

[49] Zhang DM, et al. TIGAR alleviates ischemia/reperfusion-induced autophagy and ischemic brain injury. Free Radic Biol Med. 2019;137:13–23. 30978385 10.1016/j.freeradbiomed.2019.04.002

[50] Li D, et al. 1,3,6,7-Tetrahydroxy-8-prenylxanthone ameliorates inflammatory responses resulting from the paracrine interaction of adipocytes and macrophages. Br J Pharmacol. 2018;175(10):1590–606. 29446826 10.1111/bph.14162 PMC5913410

[51] Zhang T, Linghu KG, Tan J, Wang M, Chen D, Shen Y, Wu J, Shi M, Zhou Y, Tang L, Liu L, Qin ZH, Guo B. TIGAR exacerbates obesity by triggering LRRK2-mediated defects in macroautophagy and chaperone-mediated autophagy in adipocytes. Autophagy. 2024 Aug;20(8):1741-1761. 10.1080/15548627.2024.2338576 10.1080/15548627.2024.2338576 PMC11262232 38686804

[52] Zhang T, et al. Small molecule-driven SIRT3-autophagy-mediated NLRP3 inflammasome inhibition ameliorates inflammatory crosstalk between macrophages and adipocytes. Br J Pharmacol. 2020;177(20):4645–65. 32726464 10.1111/bph.15215 PMC7520450

[53] Liu J, et al. Honokiol attenuates lipotoxicity in hepatocytes via activating SIRT3-AMPK mediated lipophagy. Chin Med. 2021;16(1):115. 34758848 10.1186/s13020-021-00528-w PMC8579168

