# Supplementary material for: Fat-targeted small molecule alleviates abnormal adipose tissue remodeling in obesity via SIRT3-driven mitophagy and inflammasome inhibition
Source: Chin Med. 2025 Dec 10;20:215. doi: 10.1186/s13020-025-01253-4 (PMC12690962; doi:10.1186/s13020-025-01253-4)
Supplement: Supplementary file 1 — Supplementary Material 1. [file 13020_2025_1253_MOESM1_ESM.docx]

**Supplementary Information**

**Fat-targeted small molecule alleviates abnormal adipose tissue remodeling in obesity via SIRT3-driven mitophagy and inflammasome inhibition**

Kegang Linghu^c,#^, Longkun Hu^a,#^, Yu-E Wang^b,c^, Yuxia Zhou^a^, Yuanyuan Wang^a^, Mingjun Shi^a^, Lirong Liu^a^, Hua Yu^d^, Lei Tang^c^, Ligen Lin^d^, Bing Guo^a,b,*^, Ai Tian^e,*^, Tian Zhang^a,b,d,*^

*^a^ Guizhou Provincial Key Laboratory of Pathogenesis and Drug Research on Common Chronic Diseases, Guizhou Medical University, Guiyang, Guizhou, 550025, China*

*^b^ Guizhou Institute of Precision Medicine, Affiliated Hospital of Guizhou Medical University, Guiyang, Guizhou, 550025, China*

*^c^ State Key Laboratory of Discovery and Utilization of Functional Components in Traditional Chinese Medicine, Guizhou Medical University, Guiyang, Guizhou, 550025, China*

*^d^ State Key Laboratory of Quality Research in Chinese Medicine, Institute of Chinese Medical Sciences, University of Macau, Macau, China*

*^e^ The Affiliated Stomatological Hospital & Stomatology of Guizhou Medical University, Guiyang, Guizhou, 550025, China*

^#^These authors contributed equally: Kegang Linghu, and Longkun Hu

*Corresponding authors: Tian Zhang, yb67520@um.edu.mo; Ai Tian, tianaident@foxmail.com; Bing Guo, guobing@gmc.edu.cn

**
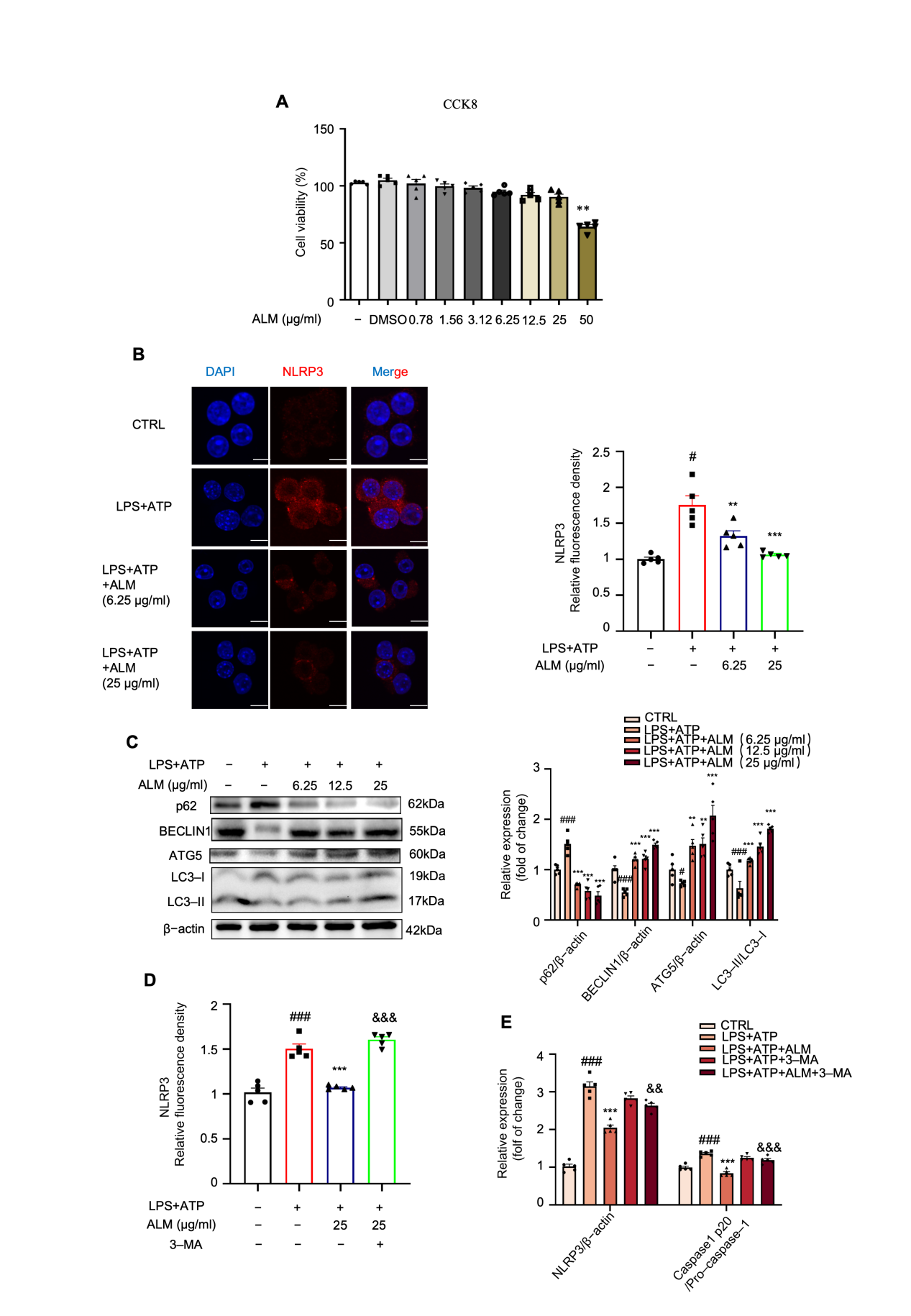
**

**Figure S1.** Raw264.7 were treated with the indicated concentration of ALM and challenged with LPS plus ATP treatment for 18 h. (A) Cell viability assay of RAW264.7 cells treated with different concentrations of ALM by CCK8 assay. (B) Immunofluorescence staining of NLRP3 in LPS plus ATP-stimulated macrophages with the indicated concentration of ALM (*n* = 5). Scale bar = 20 μm. (C) Western blot analysis of BECLIN1, ATG5, and LC3 in LPS plus ATP-stimulated macrophages with the indicated concentration of ALM (*n* = 5). β-actin was used as an internal loading control. (D) Quantification of NLRP3 immunofluorescence staining in LPS plus ATP-stimulated macrophages with the indicated concentration of ALM and 5 mM 3-MA (*n* = 5). (E) Quantifications of NLRP3 and caspase1 p20 expression in LPS plus ATP-stimulated Raw264.7 cells with the indicated concentration of ALM and 5 mM 3-MA (*n* = 5). Data are expressed as means ± SEM. ^#^ *P* < 0.05, LPS + ATP vs CTRL; * *P* < 0.05, CTRL vs. ALM, LPS + ATP + ALM vs. LPS+ATP; ^&^ *P* < 0.05, LPS + ATP + ALM vs. LPS + ATP + ALM + 3-MA.


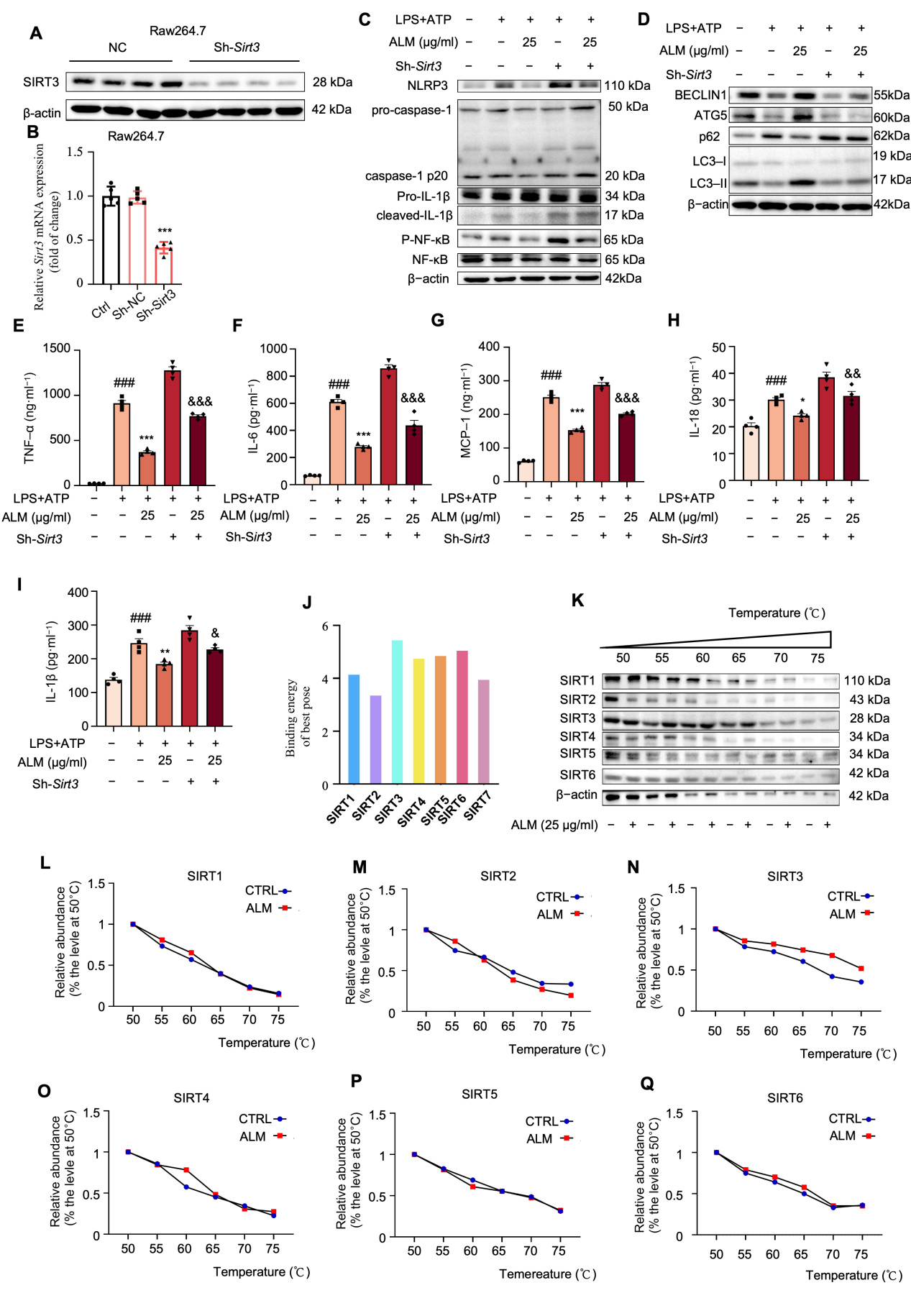


**Figure S2.** (A) Western blot analysis of SIRT3 protein in NC and Sh-*Sirt3* Raw264.7 macrophages. β-actin was used as an internal loading control. (B) Quantitative real-time PCR analysis of *Sirt3* mRNA levels in Raw264.7 cells with or without Sirt3 KD. (C) Western blot analysis of NF-κB, NLRP3, caspase-1, and IL-1β in LPS plus ATP and ALM-treated cells with or without Sirt3 KD (*n* = 3). (D) Western blot analysis of BECLIN1, ATG5, p62, and LC3 in LPS plus ATP and ALM-treated cells with or without Sirt3 KD (*n* = 3). (E-H) Measurement of the TNF-α, IL-6, MCP-1, and IL-18 levels in the Raw264.7 cell culture medium by ELISA kits (*n* = 4). (I) Measurement of the IL-1β level of the Raw264.7 cell lysates by ELISA kits (*n* = 4).(J) Docking results of the binding between ALM and SIRT1 (PDB ID: 4ZZH), SIRT2 (PDB ID: 1J8F), SIRT3(PDB ID: 5H4D), SIRT4 (PDB ID: 5OJN), SIRT5 (PDB ID: 8GBL), SIRT6 (PDB ID: 5X16), and SIRT7 (PDB ID: 5IQZ). Cluster analysis of the docked conformations of ALM. A tolerance of 2.0 Å was used. (K) Cellular thermal shift assay (CETSA) was performed on RAW264.7 cells after the treatment with or without ALM (25 μg·ml^-1^) for 18 h (*n* = 4). (L-Q) Quantification of CETSA for SIRT1-SIRT6 *(n* = 4). β-actin was used as an internal loading control. Data were normalized to the mean value of each protein level at 50℃. Data are expressed as means ± SEM. ^#^ *P* < 0.05 and ^###^ *P* < 0.001, LPS + ATP vs. CTRL; * *P* < 0.05 ** *P* < 0.01 and *** *P* < 0.001, CTRL or Sh-NC vs. Sh-*Sirt3*; LPS + ATP+ALM vs. LPS + ATP; ^&^ *P* < 0.05 and ^&&^ *P* < 0.01, LPS + ATP + ALM vs. LPS + ATP + ALM + Sh-*Sirt3*.

**
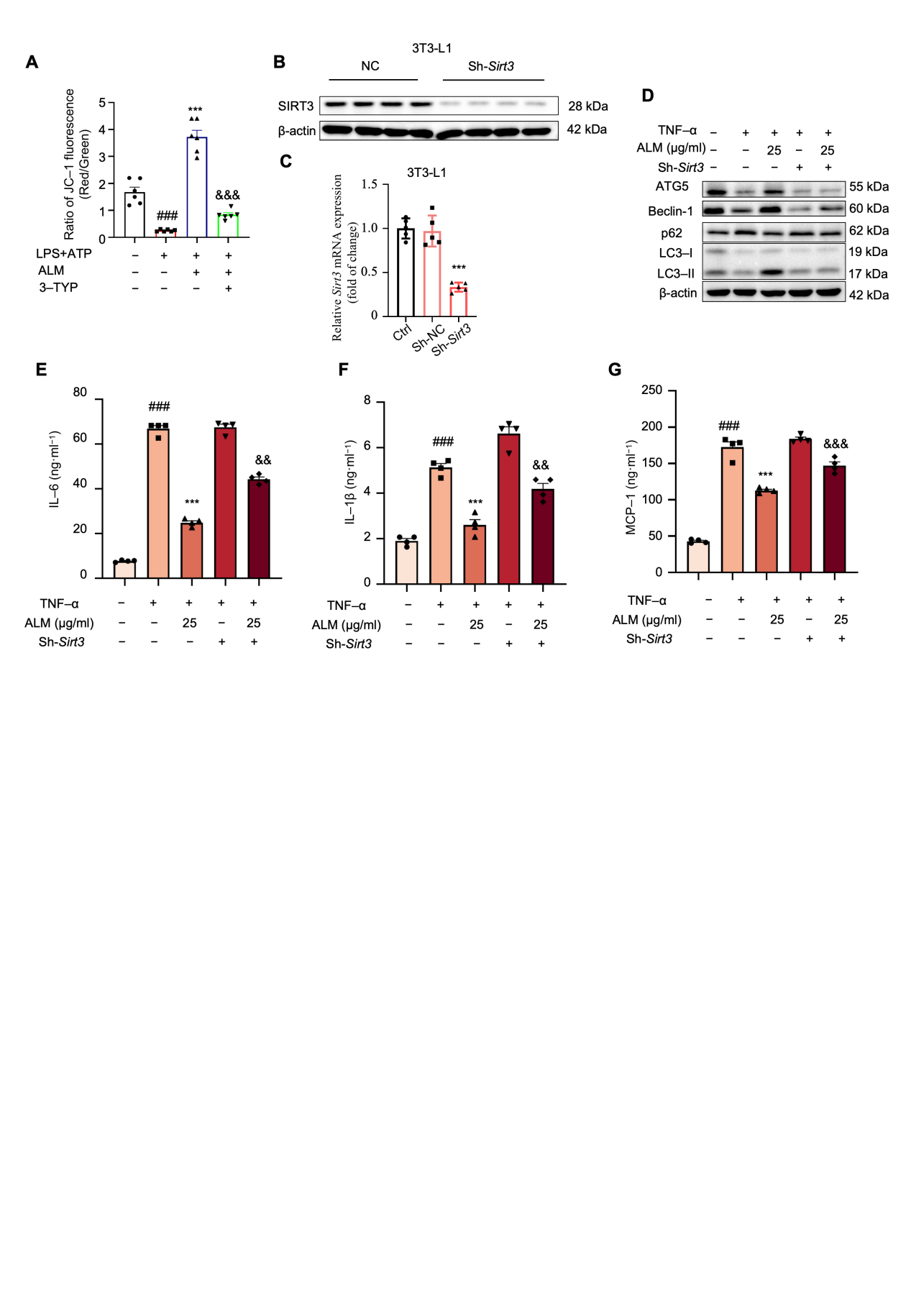
**

**Figure S3.** (A) Quantification of JC-1 staining in LPS plus ATP-stimulated Raw264.7 with indicated concentration of ALM and 50 μM 3-TYP (n = 6). (B) Western blot analysis of SIRT3 protein in NC and Sh-*Sirt3* 3T3-L1 adipocytes. β-actin was used as an internal loading control. (C) Quantitative real-time PCR analysis of *Sirt3* mRNA levels in 3T3-L1 cells with or without Sirt3 KD. (D) Western blot analysis of BECLIN1, ATG5, p62, and LC3 in TNF-α and ALM-treated cells with or without Sirt3 KD (*n* = 3). (E-G) Measurement of the IL-6, IL-1β, and MCP-1 levels in the 3T3-L1 cell culture medium by ELISA kits (*n* = 4). Data are expressed as means ± SEM. ^#^ *P* < 0.05, CTRL vs. LPS + ATP; TNF-α vs. CTRL ^**^ *P* < 0.01 and ^***^ *P* < 0.001, LPS + ATP +A LM vs. LPS + ATP; CTRL or Sh-NC vs. Sh-*Sirt3*; TNF-α + ALM vs. TNF-α; ^&^ *P* < 0.05 and ^&&^ *P* < 0.01, LPS + ATP + ALM + 3-TYP vs. LPS+ATP+ALM; TNF-α + ALM vs. TNF-α + ALM + Sh-*Sirt3*;

**
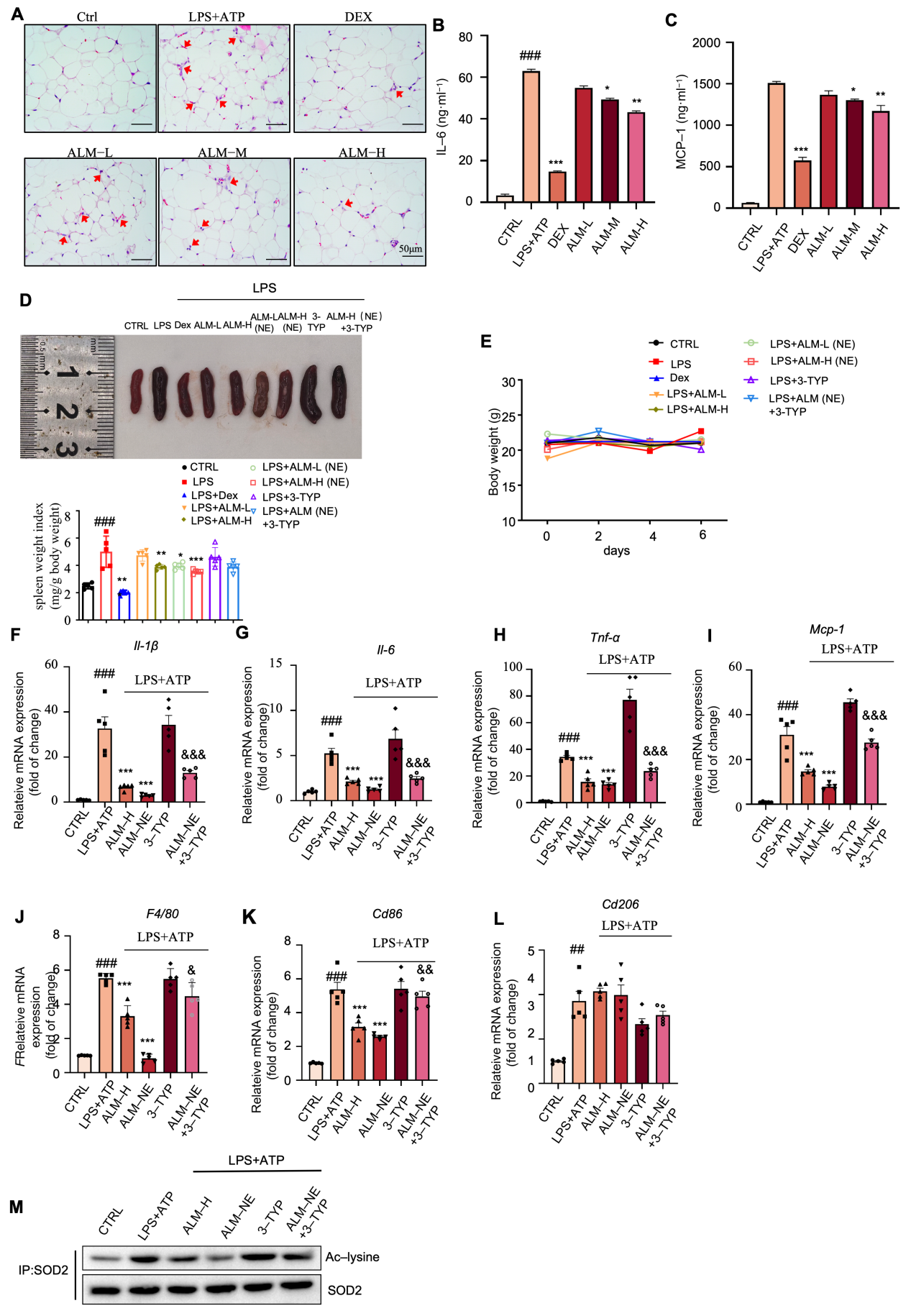
**

**Figure S4.** (A) H&E staining of eWAT (n = 5). The levels of IL-6 (B) and MCP-1 (C) were determined by ELISA kits (*n* = 5). (D) Representative images of acute edema of the spleen after LPS injection, and ALM dose-dependently attenuated the degree of edema and the spleen weight index (*n* = 5). (E) The body weights in different groups of mice. (F-I) Quantitative real-time PCR analysis of *Il-1β*, *Il-6*, *Tnf-α,* and *Mcp-1* mRNA levels in adipose tissue from LPS plus ATP-treated mice (*n* = 5). (J-L) Quantitative real-time PCR analysis of *F4/80, Cd86, and Cd206* mRNA levels in peritoneal macrophages from LPS plus ATP-treated mice. (M) Co-IP assay indicates the level of acetylated SOD2 in adipose tissue from LPS plus ATP-treated mice. Data are expressed as means ± SEM. ^#^ *P* < 0.05, HFD vs. CTRL; * *P* < 0.05, HFD vs ALM-H or ALM-NE; ** *P* < 0.01, HFD vs ALM-H or ALM-NE; *** *P* < 0.001, HFD vs ALM-H or ALM-NE.


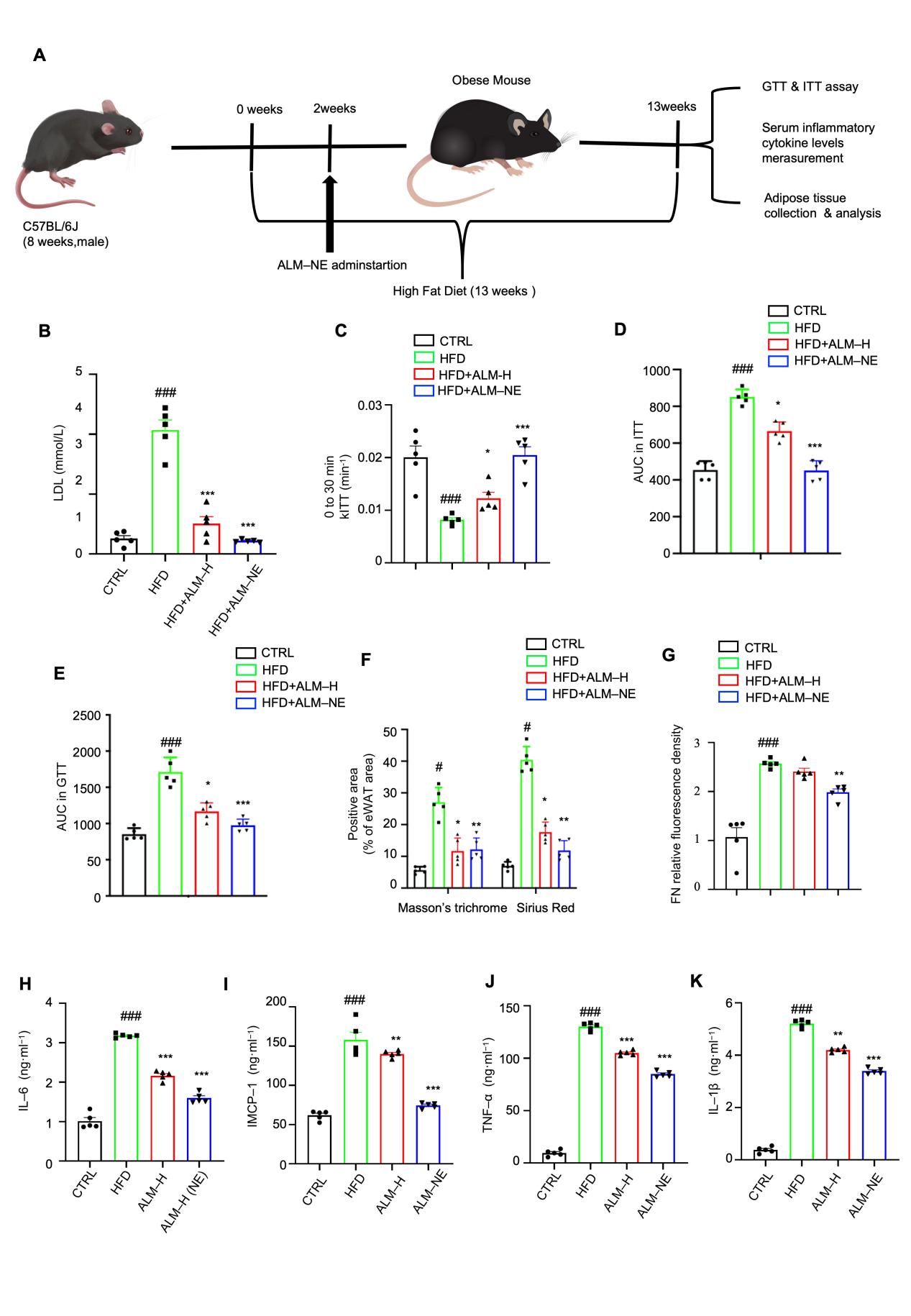


**Figure S5.** (A) The experimental procedure of HFD-induced obesity. (B) The serum LDL level in different groups of HFD mice (*n* = 5). (C) The rates of glycemia decay (kITT) from 0 to 30 min in the ITT. (D-E) Area under curve (AUC) in GTT and ITT of HFD mice (*n* = 5). (F) Statistical graphs of Masson’s trichrome and Sirius red staining (*n = 5*). (G) FN fluorescence density was assayed (*n* = 5). (H-K) The pro-inflammatory cytokines (IL-1β, TNF-α, IL-6, and MCP-1) in serum from HFD and indicated treatment mice were determined by ELISA kits (*n = 5*). Data are expressed as means ± SEM (*n = 5*). Data are expressed as means ± SEM. ^#^ *P* < 0.05, HFD vs. CTRL; * *P* < 0.05, HFD vs ALM-H or ALM-NE; ** *P* < 0.01, HFD vs ALM-H or ALM-NE; *** *P* < 0.001, HFD vs ALM-H or ALM-NE.
